# Supplementary material for: White matter myelination during early infancy is linked to spatial gradients and myelin content at birth
Source: Nat Commun. 2022 Feb 22;13:997. doi: 10.1038/s41467-022-28326-4 (PMC8863985; doi:10.1038/s41467-022-28326-4)

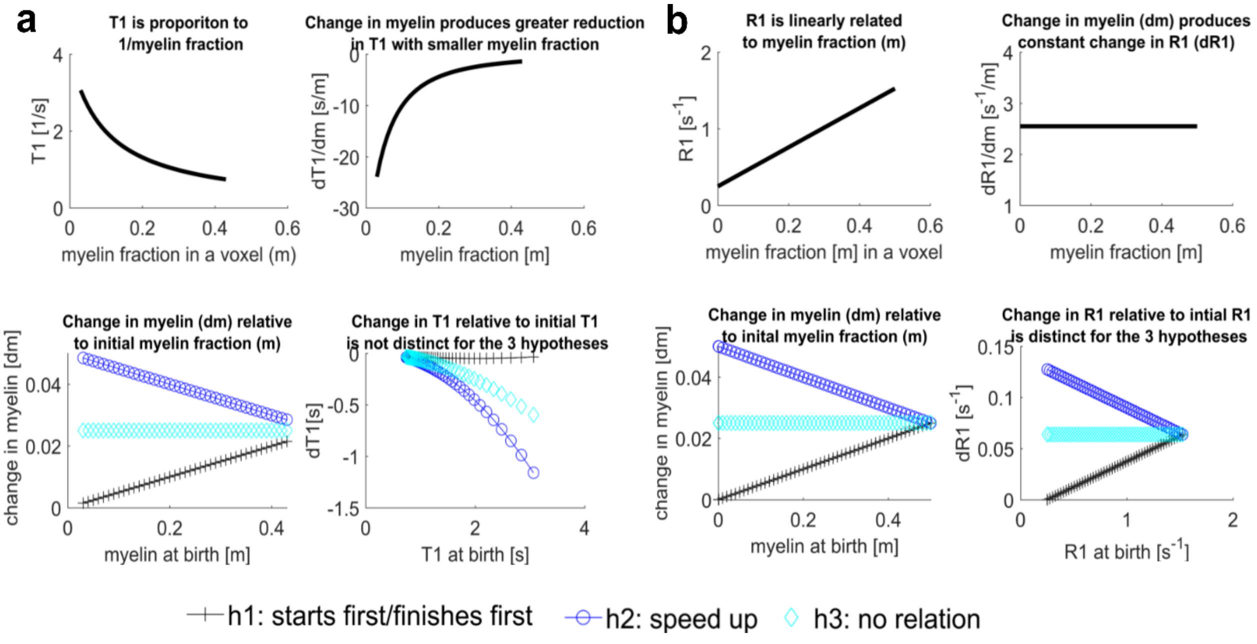

Supplementary Figure 1. Quantitative MRI measures of R1 (b) but not T1 (a) are linearly related to myelin content. As such, R1 is a suitable metric to distinguish between developmental hypotheses.

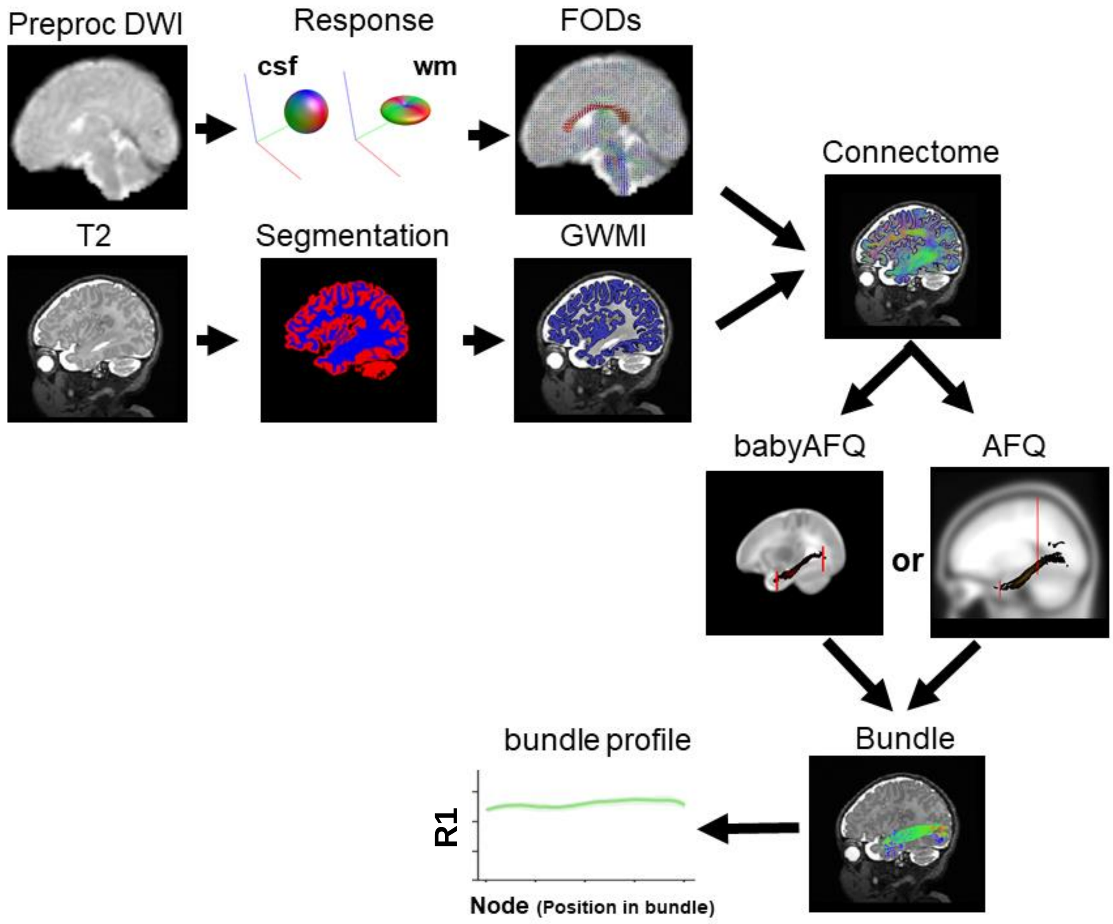

Supplementary Figure 2. A schematic representation of our processing pipeline. DMRI data is preprocessed and fiber density functions (FODs) are generated separately for white matter and CSF using multi-shell/multi-tissue constrained spherical deconvolution (CSD). A tissue segmentation is generated from anatomical data and used for anatomically constrained tractography (ACT). Seeds for tractography are placed at the gray/white matter interface (GWMI) and whole brain connectomes with 2 million streamlines are created for each infant and timepoint. Bundles are delineated from the whole brain connectome using either babyAFQ, which was developed here and optimized for infant data or AFQ, which was developed from adult data and serves as a benchmark. R1 development is then evaluated across the length of each bundle identified with babyAFQ.

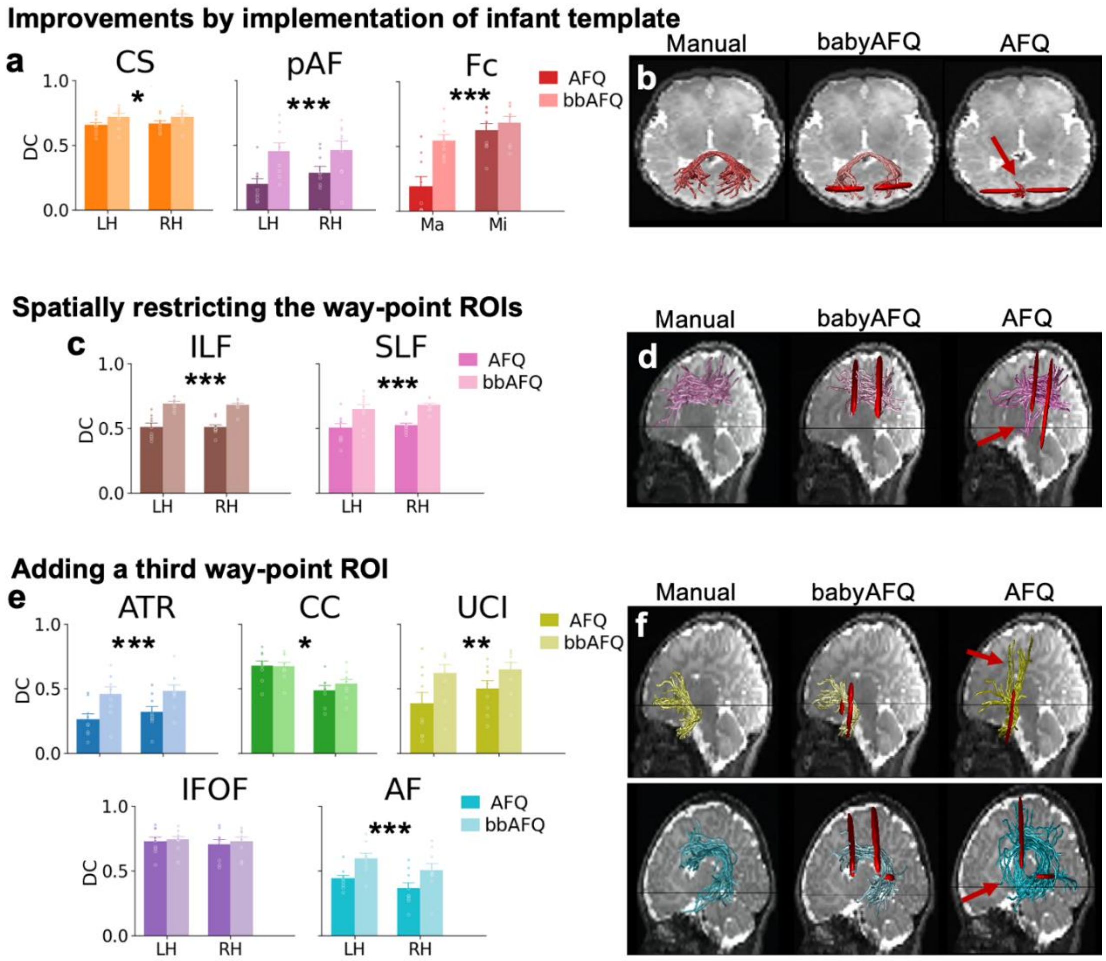

Supplementary Figure 3. Adapting AFQ for the infant brain (babyAFQ) improves the identification of white matter bundles in newborns. Infant-specific adaptations included the usage of an infant template brain for definition of way-point ROIs for all tracts, spatially restricted way-point ROIs for the ILF and SLF and the introduction of a third way-point ROIs for "curvy" bundles. a,c,e: Bar graphs compare AFQ and babyAFQ to manually delineated "gold-standard" bundles in newborns (N=9). Higher dice coefficients (DCs) indicate more spatial overlap with manual tracts. Repeated measures ANOVAs (factors: AFQ and hemisphere / anteriority) show main effect of AFQ for most bundles: CS: $F(8)=11.45$, $p=0.01$, pAF: $F(8)=56.82$, $p=0.0001$, Fc: $F(8)=20.80$, $p=0.0007$, ILF: $F(8)=159.63$, $p<0.0001$, SLF: $F(8)=99.46$, $p<0.0001$, ATR: $F(8)=123.11$, $p<0.0001$, CC: $F(8)=9.70$, $p=0.01$, UCI: $F(8)=21.34$, $p=0.008$, IFOF: $F(8)=1.06$, $p=0.33$, AF: $F(8)=36.25$, $p=0.0003$. Bars show mean DCs ± SEM. *$p<0.05$, **$p<0.01$, ***$p<0.001$. Circles indicate individual infants' data. b,d,f: Examples of bundles delineated manually as well as with AFQ and babyAFQ presented in individual subject's left hemisphere. Abbreviations: LH: left hemisphere, RH: right hemisphere, CS: cortico-spinal tract, pAF: posterior arcuate fasciculus, Fc: forceps (Ma=forceps major; Mi=forceps minor), ILF: inferior longitudinal fasciculus, SLF: superior longitudinal fasciculus, ATR: anterior thalamic radiation, CC: cingulum cingulate, UCI: uncinate fasciculus, IFOF: inferior frontal occipital fasciculus, AF: arcuate fasciculus.

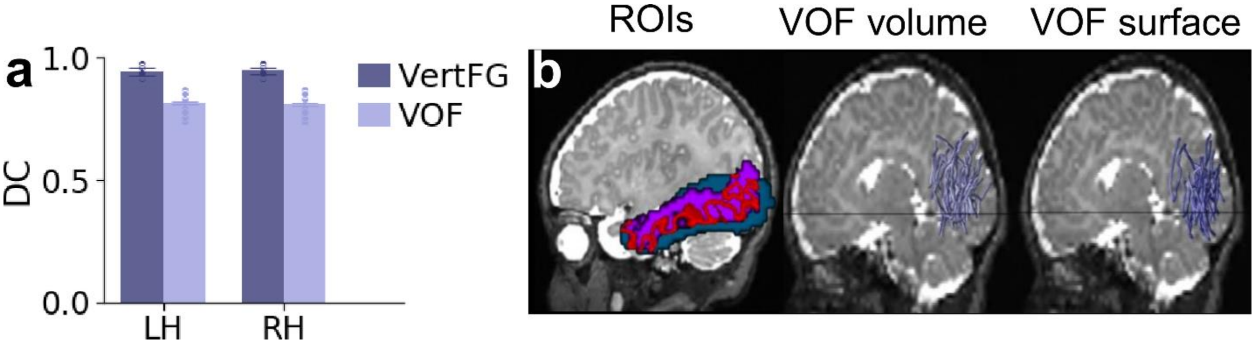

Supplementary Figure 4. Tracts identified as being part of the VOF are similar regardless of whether surface or volume waypoint ROIs are used. While AFQ uses surface ROIs to define the VOF, babyAFQ offers both surface and volume ROIs to define this bundle, as to date it is difficult to generate cortical surfaces in infants. a. Bar graph shows DC for tracts identified using surface ROIs and volume ROIs. Dark bars indicate overlap of all vertical tracts and light bars indicate overlap of the VOF bundle identified with both approaches. Bars show mean DC ± SEM separately for both hemispheres. Circles indicate individual infants' data. b. ROIs and identified VOF bundles in an example subject. Left panel: purple outline shows an example surface ROI, blue box shows an example volume ROI and red outline indicates gray-white matter interface within volume ROI. Middle panel: VOF identified using a volume ROI. Right panel: VOF identified using a surface ROI. Abbreviations: LH: left hemisphere, RH: right hemisphere, VertFG: vertical fiber group, VOF: vertical occipital fasciculus.

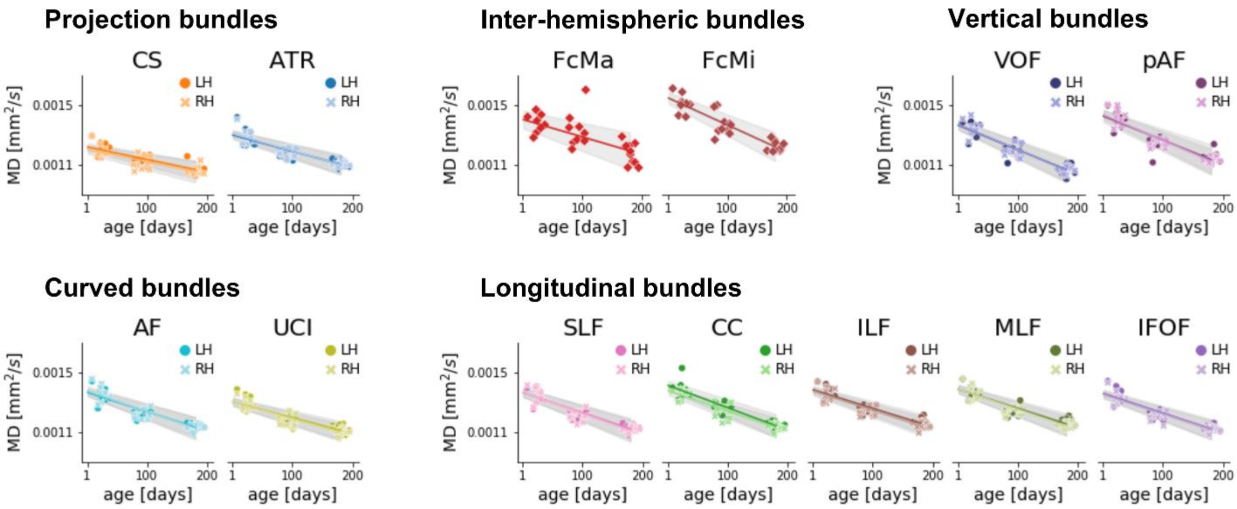

Supplementary Figure 5. MD of white matter bundles linearly decreases from birth to 6 months of age. Mean MD of each bundle as a function of age in days. Each point is a participant; markers indicate hemisphere; lines indicate LMM prediction; lines for both hemispheres fall on top of each other; gray shaded regions indicate 95% confidence intervals. Abbreviations: MD: mean diffusivity, LH: left hemisphere, RH: right hemisphere, CS: cortico-spinal tract, ATR: anterior thalamic radiation, FcMa: forceps major; FcMi: forceps minor, VOF: vertical occipital fasciculus, pAF: posterior arcuate fasciculus, AF: arcuate fasciculus, UCI: uncinate fasciculus, SLF: superior longitudinal fasciculus, CC: cingulum cingulate, ILF: inferior longitudinal fasciculus, MLF: middle longitudinal fasciculus, IFOF: inferior frontal occipital fasciculus.

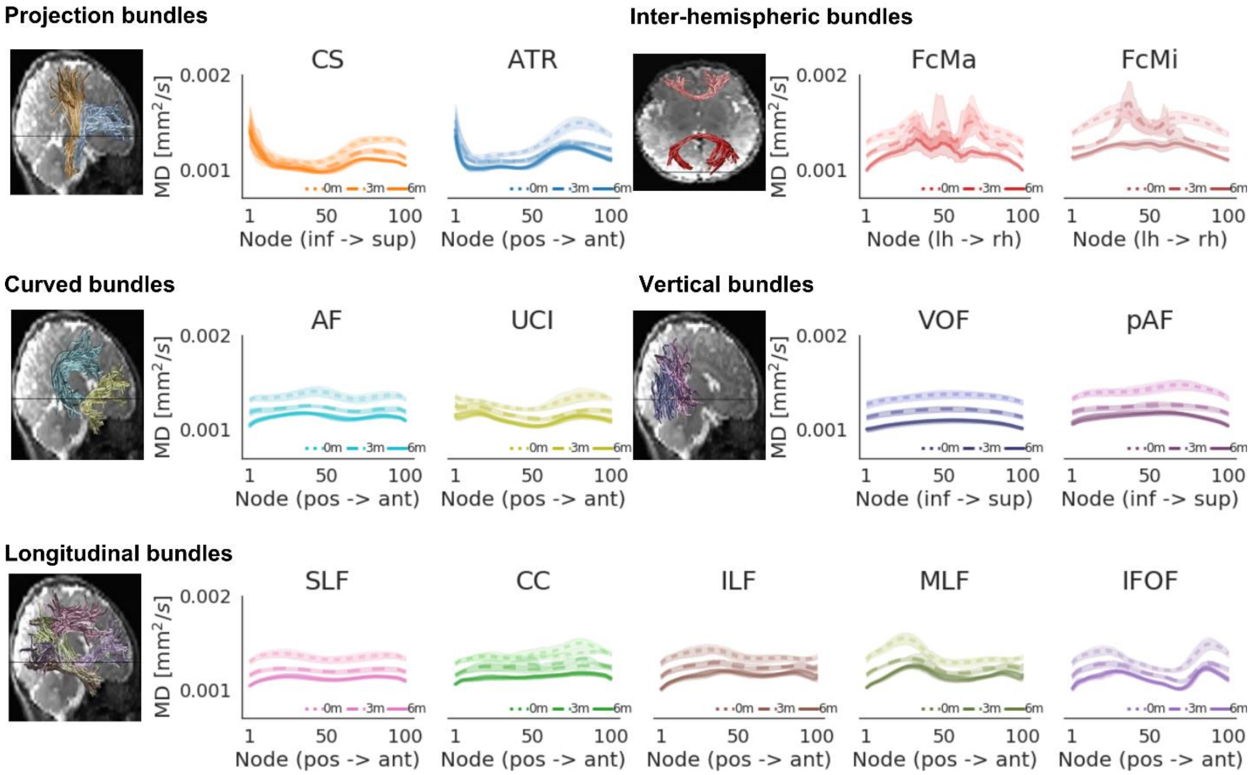

Supplementary Figure 6. Development of MD along each bundle. Mean MD across infants is displayed in both hemispheres (lines for the two hemispheres fall on top of each other) along the length of each bundle in newborns (0m, dotted line), 3-months-olds (3m, dashed line), and 6-months-olds (6m, solid line). Shaded regions: 95% confidence intervals. Left panels show the bundles in a representative newborn. Abbreviations: MD: mean diffusivity, CS: cortico-spinal tract, ATR: anterior thalamic radiation, FcMa: forceps major; FcMi: forceps minor, VOF: vertical occipital fasciculus, pAF: posterior arcuate fasciculus, AF: arcuate fasciculus, UCI: uncinate fasciculus, SLF: superior longitudinal fasciculus, CC: cingulum cingulate, ILF: inferior longitudinal fasciculus, MLF: middle longitudinal fasciculus, IFOF: inferior frontal occipital fasciculus.

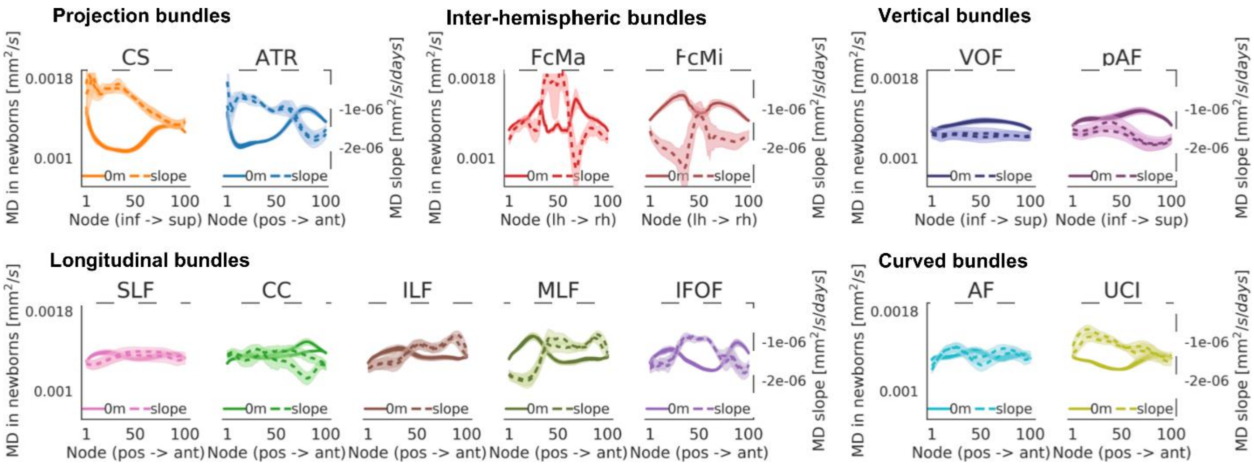

Supplementary Figure 7. MD development rate varies along the length of each bundle. Each panel jointly shows the measured MD in newborns (left y axis, solid line) and the slope of MD development (right y axis, dashed line) at each node along the bundle. Faster development (more negative slope) corresponds to lower values of dashed lines. Higher MD in newborns correspond to higher values in solid lines. Lines from both hemispheres are presented separately but fall on top of each other. Shaded regions indicate standard error of measured MD in newborns or slope of MD development, respectively. Abbreviations: MD: mean diffusivity, CS: cortico-spinal tract, ATR: anterior thalamic radiation, FcMa: forceps major; FcMi: forceps minor, VOF: vertical occipital fasciculus, pAF: posterior arcuate fasciculus, AF: arcuate fasciculus, UCI: uncinate fasciculus, SLF: superior longitudinal fasciculus, CC: cingulum cingulate, ILF: inferior longitudinal fasciculus, MLF: middle longitudinal fasciculus, IFOF: inferior frontal occipital fasciculus.

Supplementary Figure 8. BabyAFQ successfully identifies all bundles in all infants and timepoints. On the following pages, we show each individual's bundles identified with babyAFQ as well as AFQ and manually (where applicable). Data is sorted by bundle. Bundles identified with babyAFQ show the expected spatial extent and trajectory across all participants.

Left Anterior Thalamic Radiation
Newborn

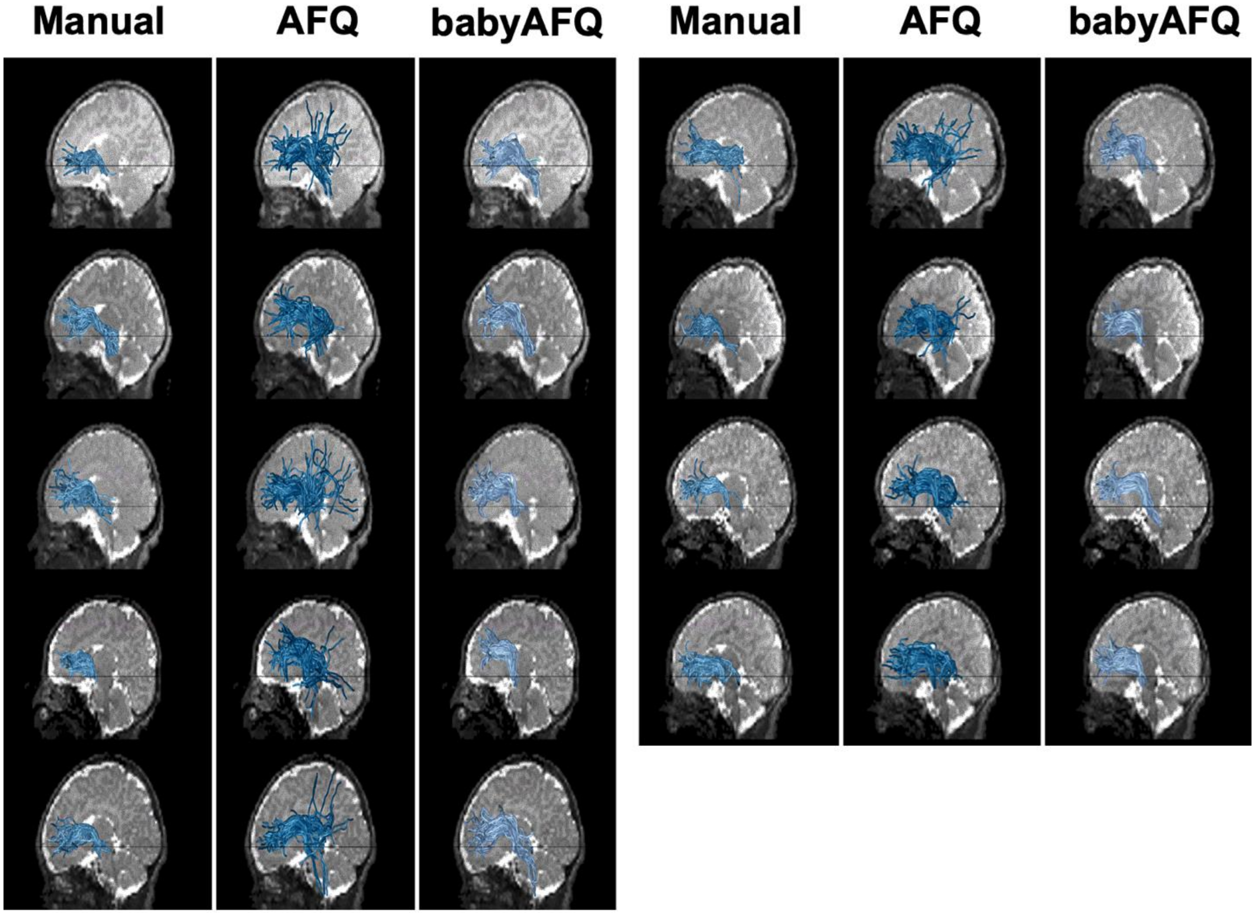

Left Anterior Thalamic Radiation

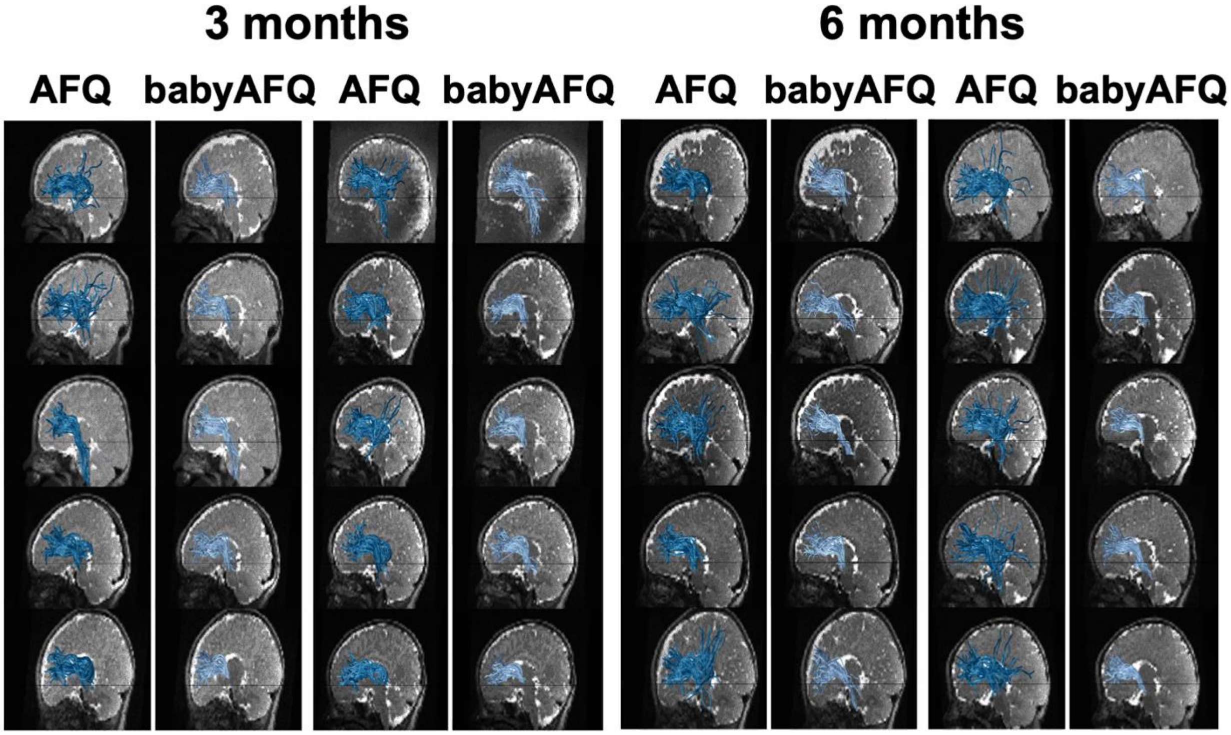

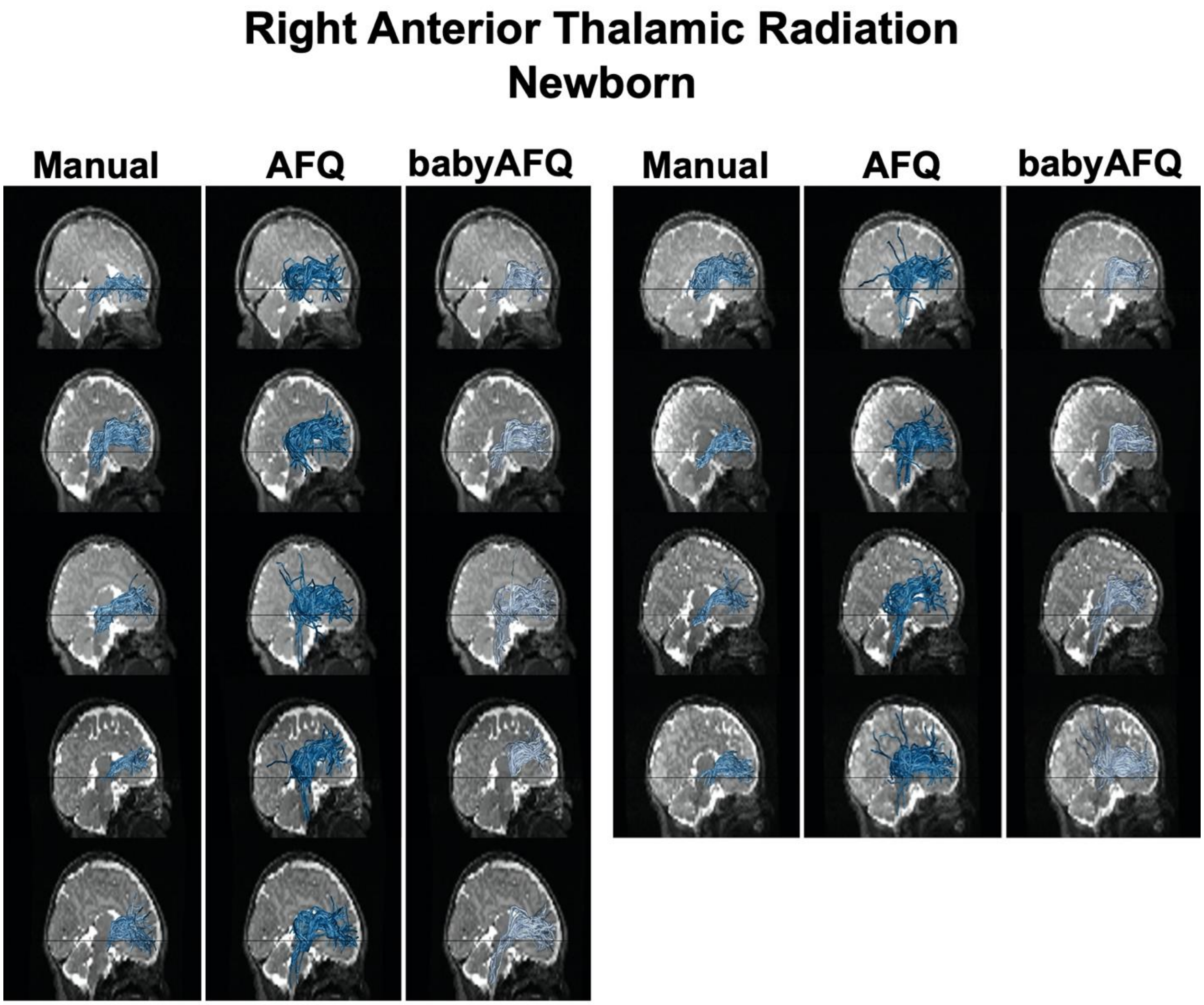
Right Anterior Thalamic Radiation
Newborn
Manual
AFQ
babyAFQ
Manual
AFQ
babyAFQ

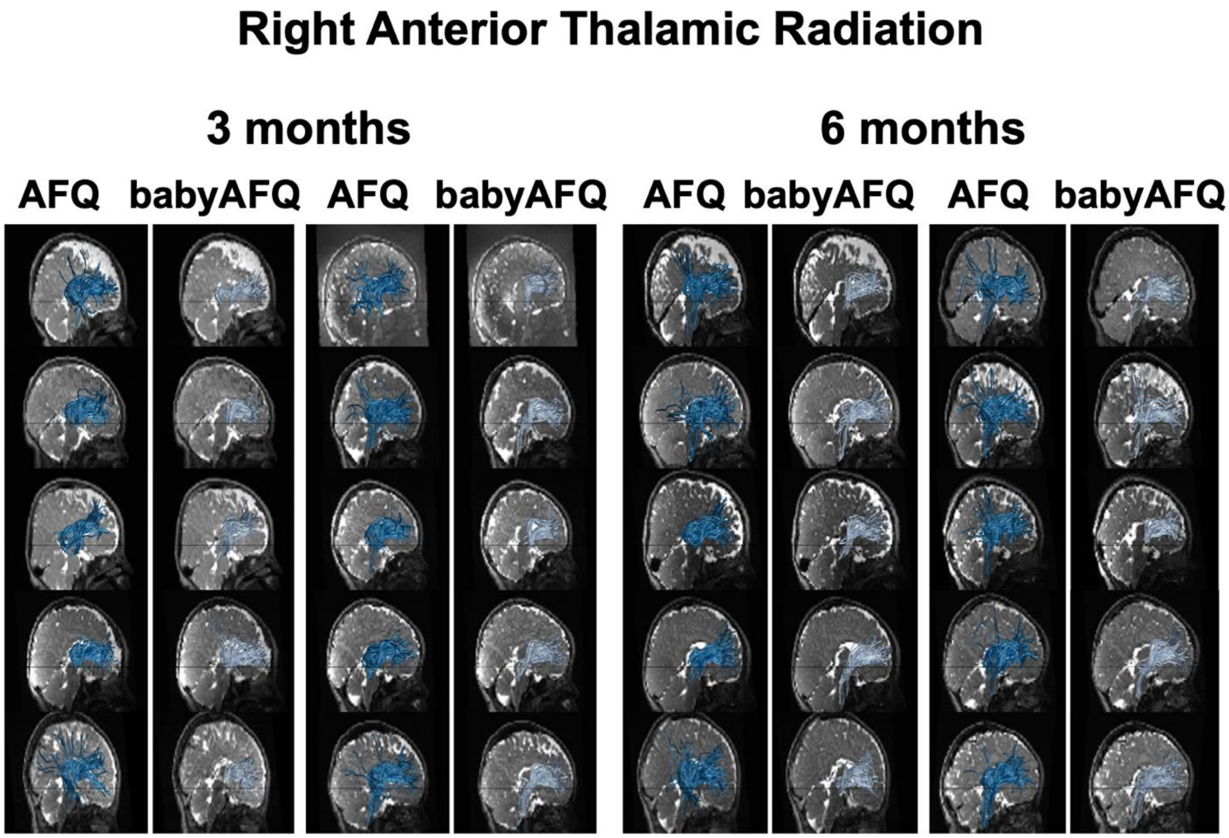
Right Anterior Thalamic Radiation
3 months
6 months
AFQ
babyAFQ
AFQ
babyAFQ
AFQ
babyAFQ
AFQ
babyAFQ

Left Corticospinal Tract
Newborn

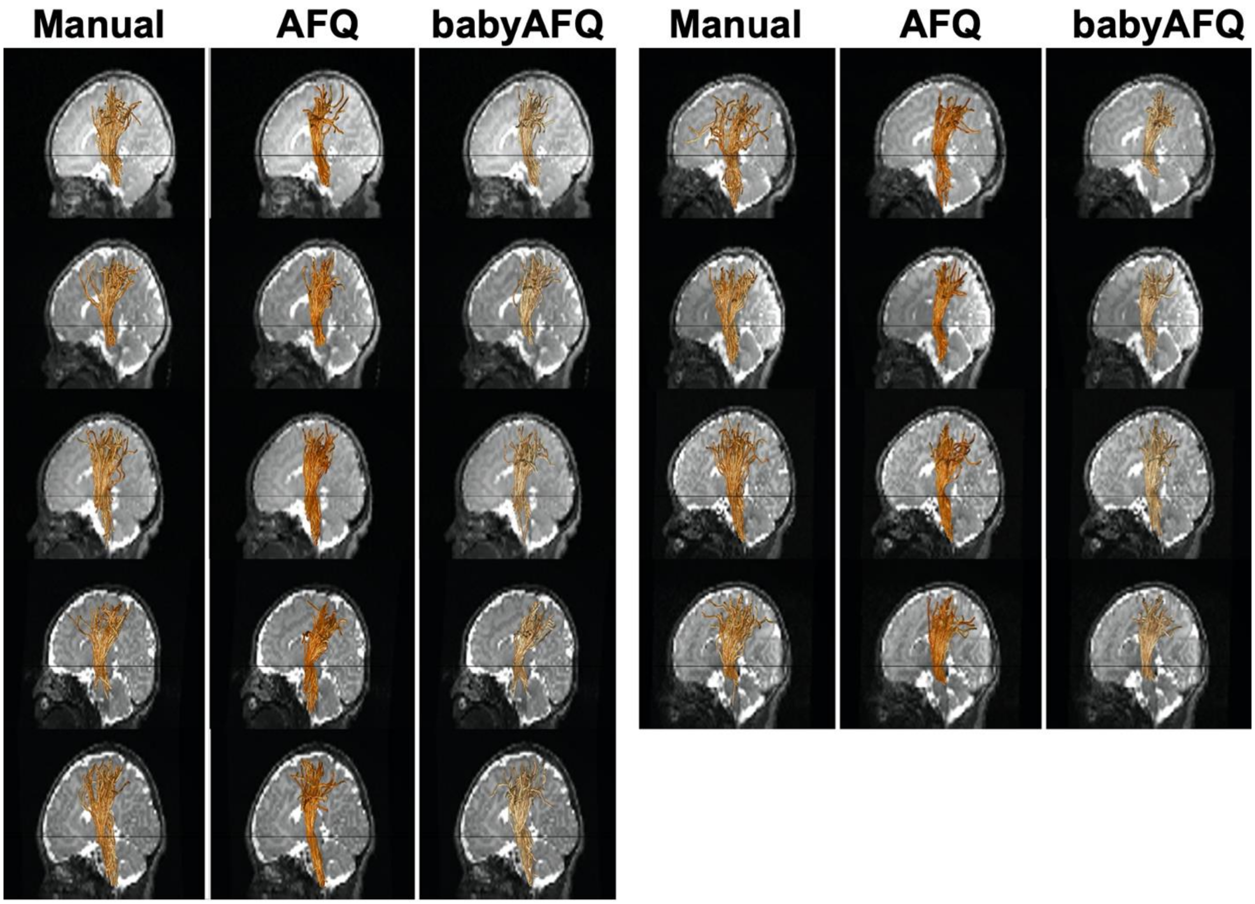

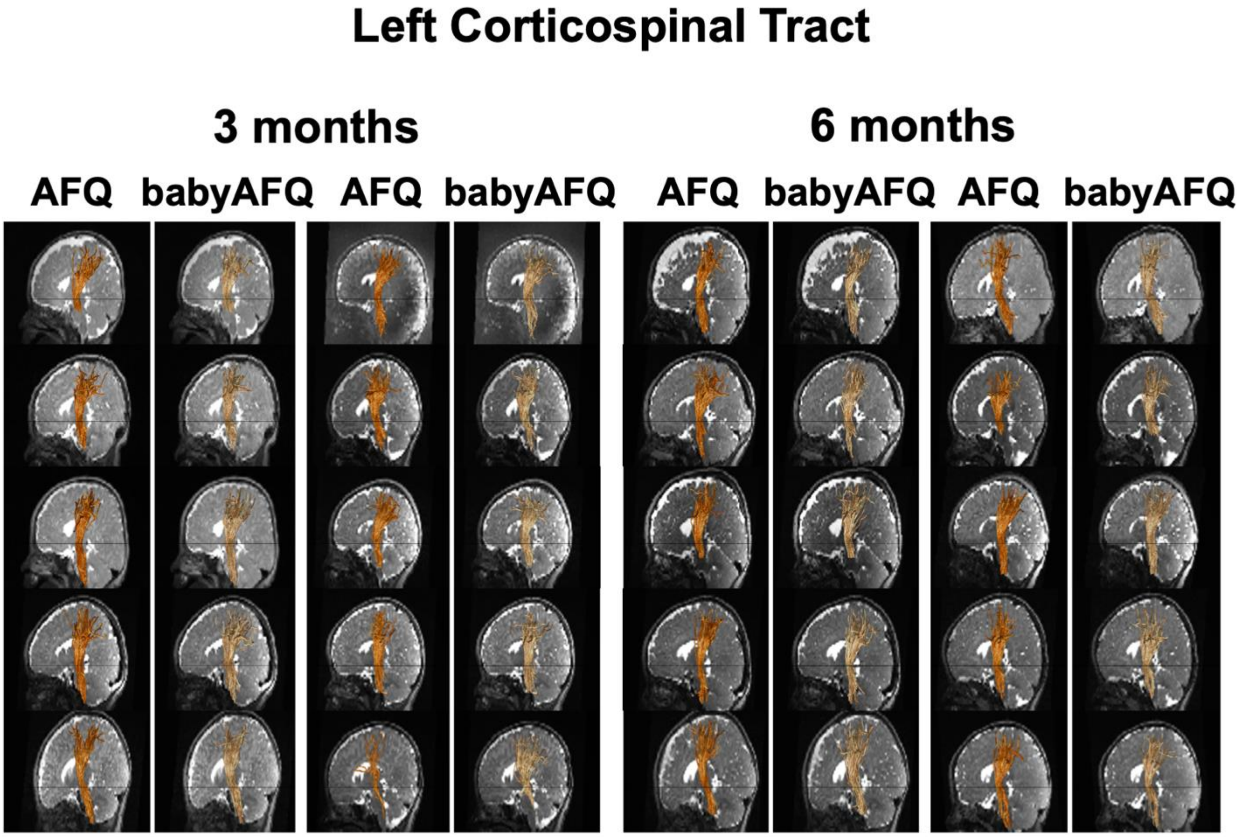
Left Corticospinal Tract
3 months
6 months
AFQ
babyAFQ
AFQ
babyAFQ
AFQ
babyAFQ
AFQ
babyAFQ

Right Corticospinal Tract
Newborn

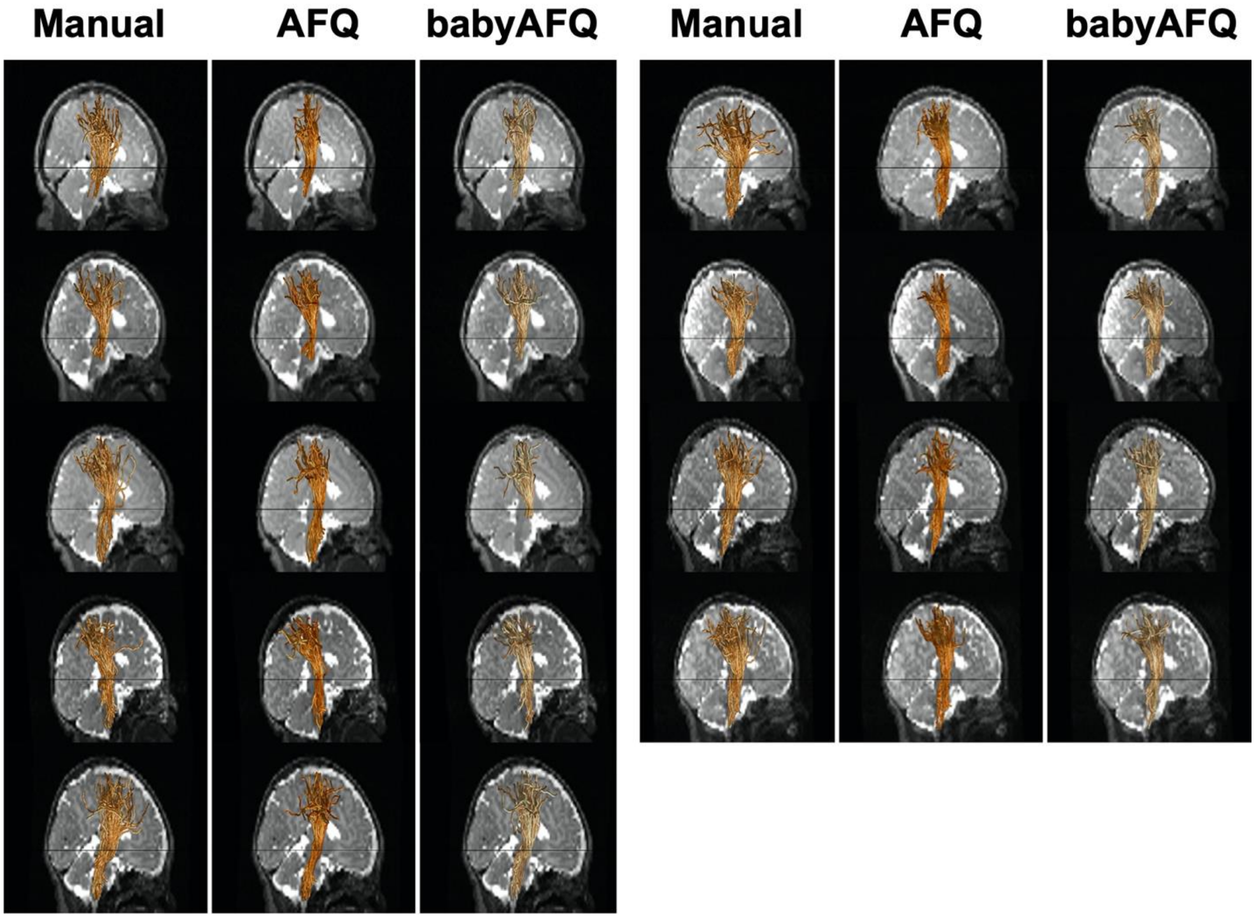

Right Corticospinal Tract

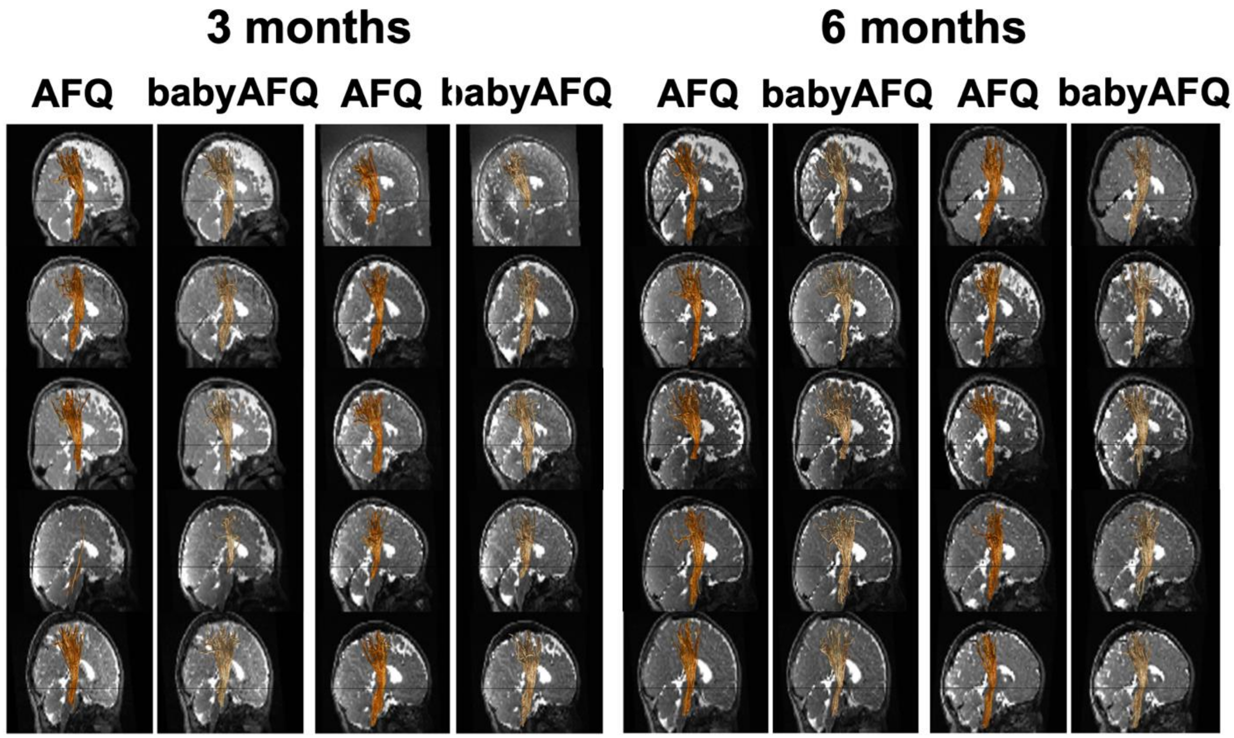

Left Cingulum Cingulate
Newborn

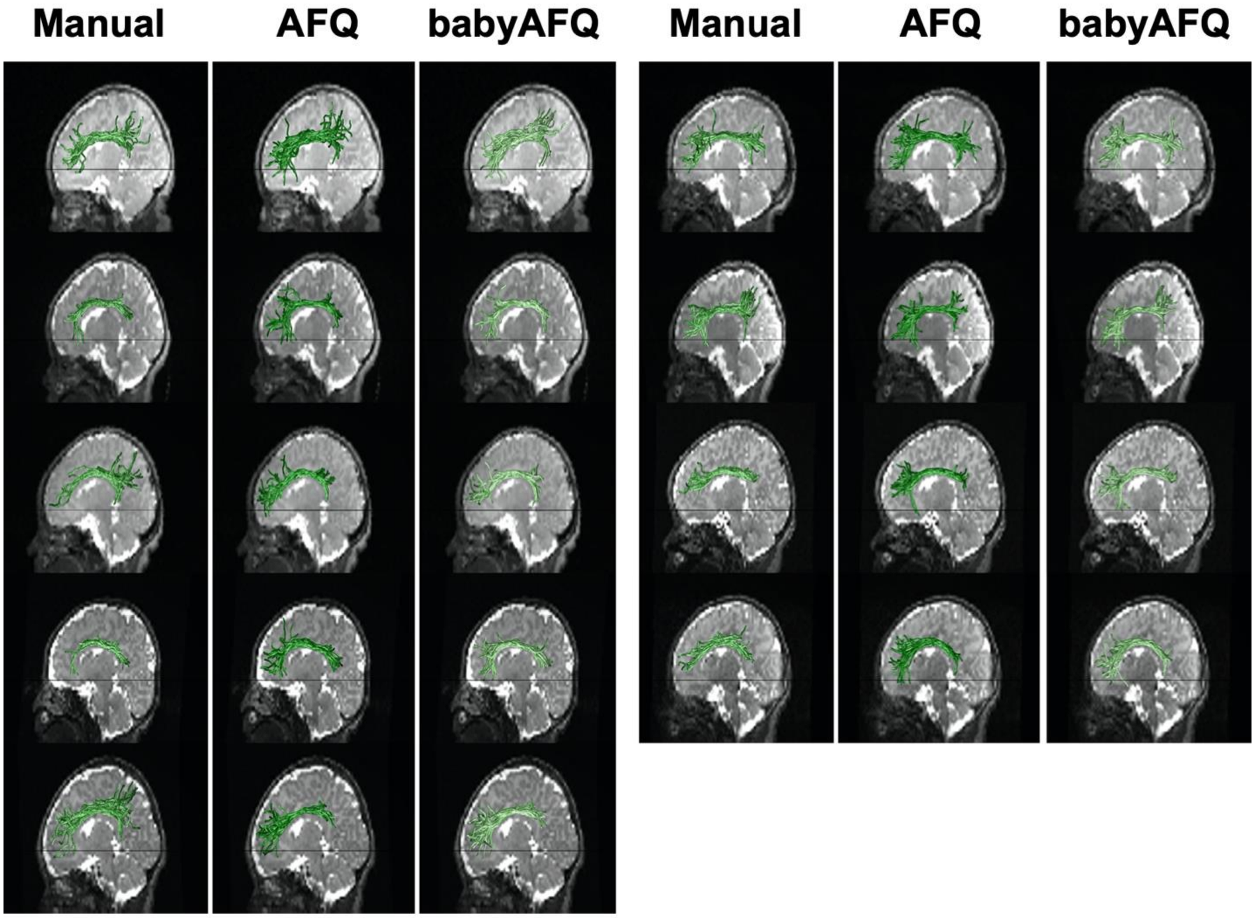

Left Cingulum Cingulate

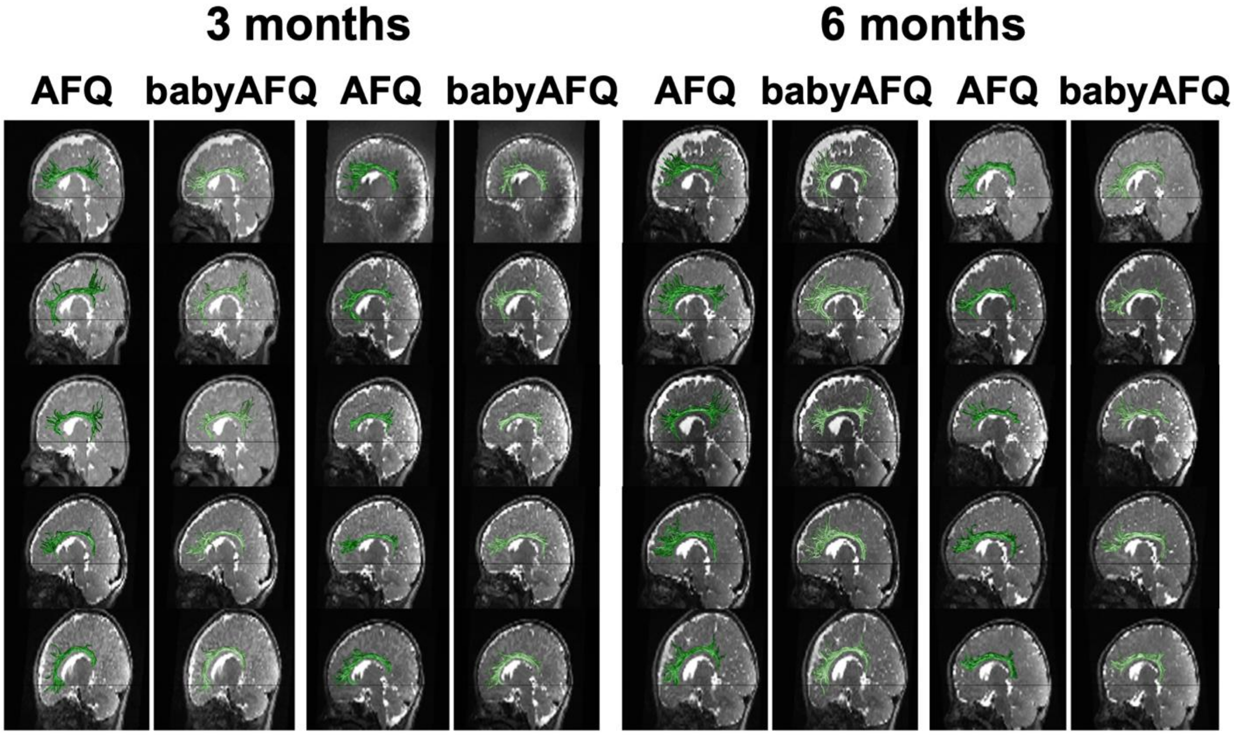

Right Cingulum Cingulate
Newborn

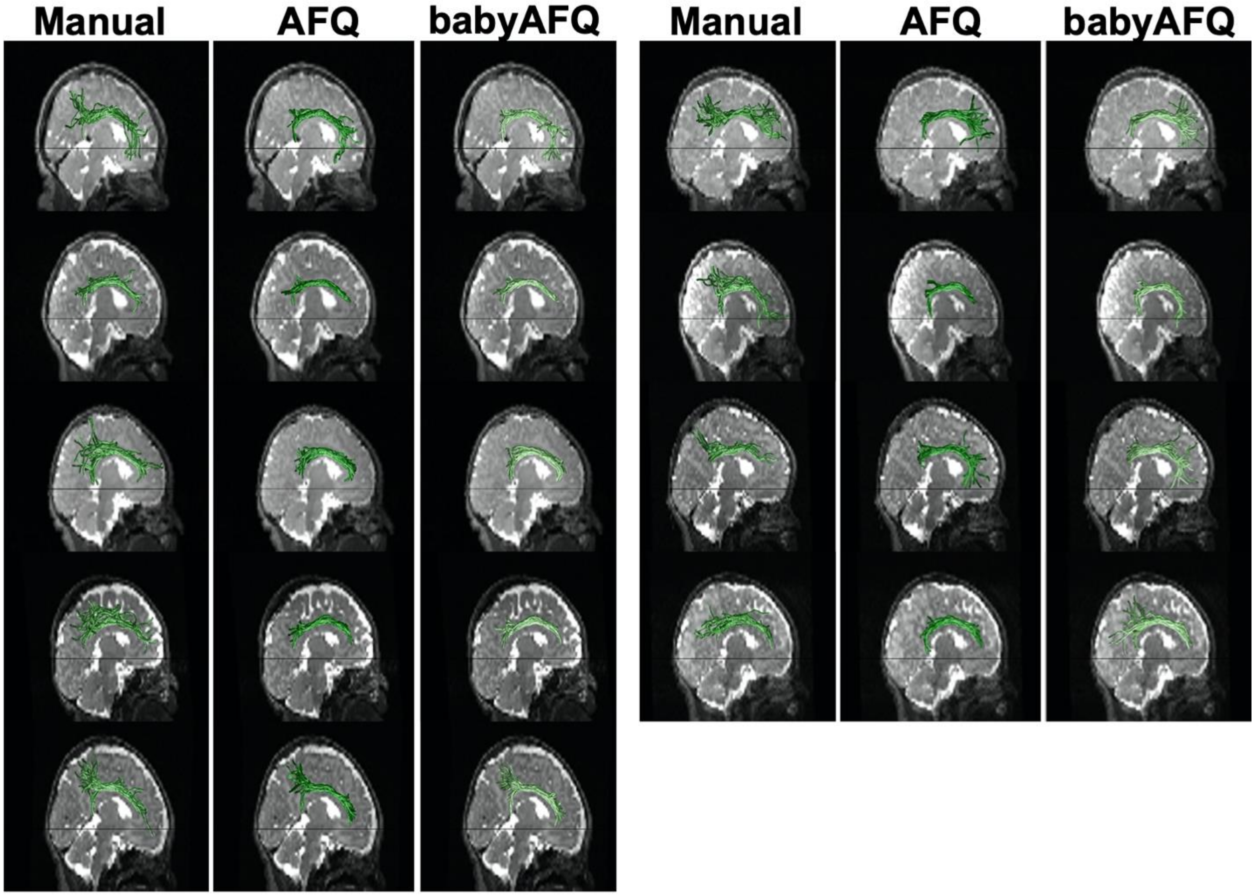

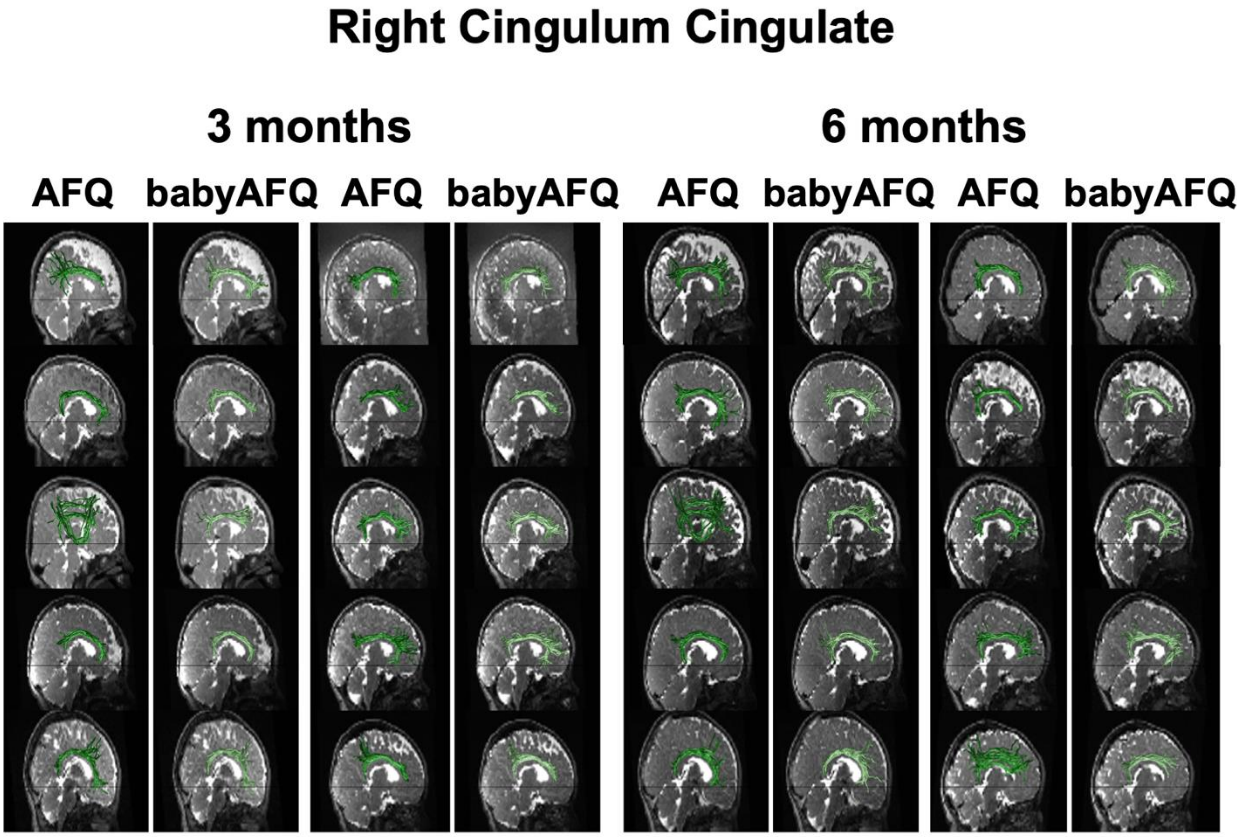
Right Cingulum Cingulate
3 months
6 months
AFQ
babyAFQ
AFQ
babyAFQ
AFQ
babyAFQ
AFQ
babyAFQ

Forceps Major
Newborn

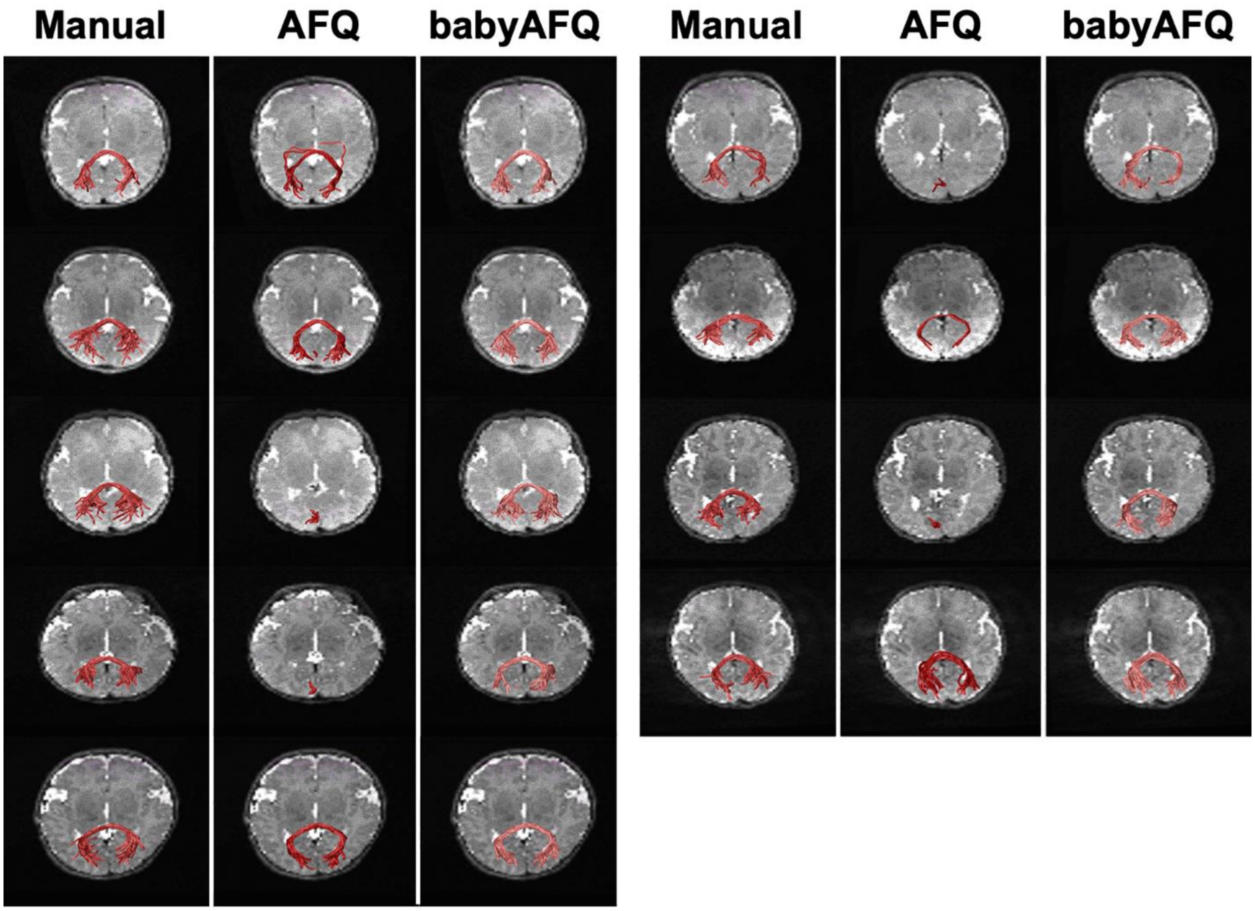

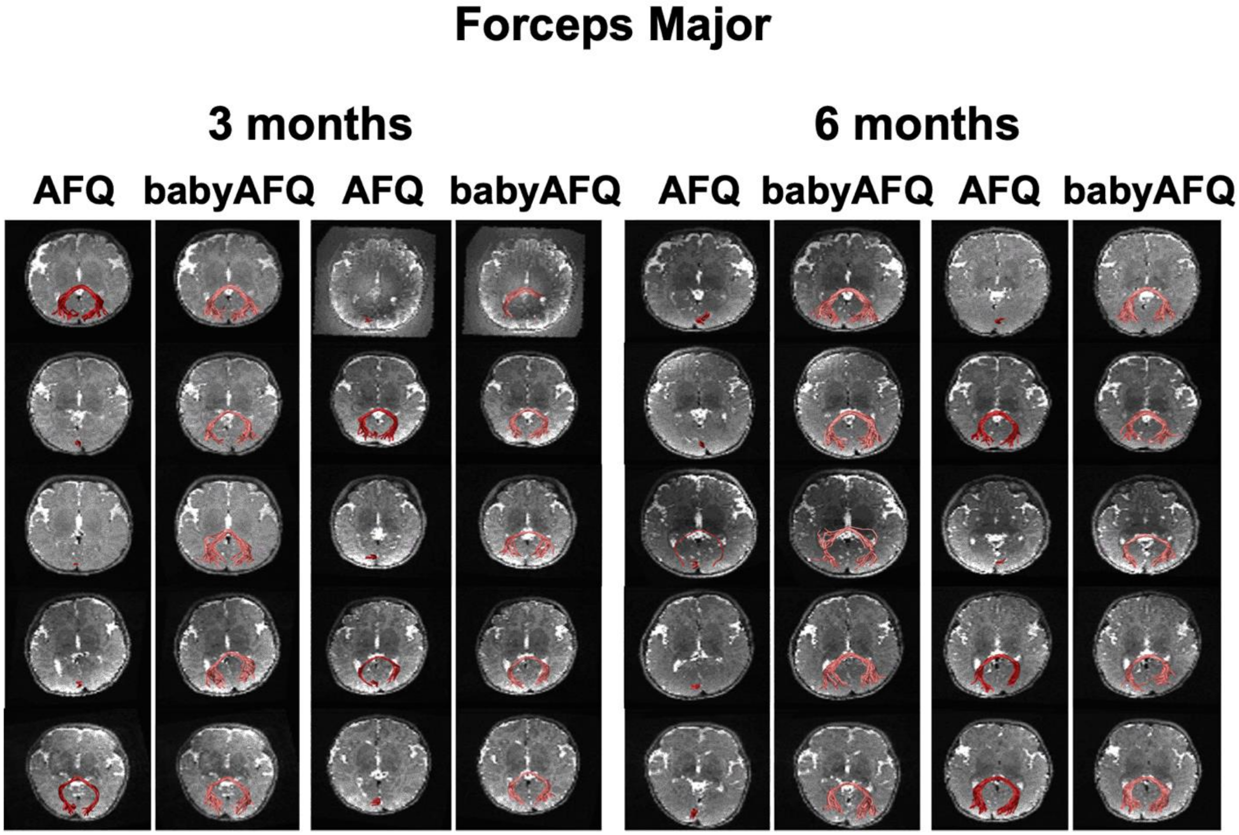

Forceps Major
3 months
6 months
AFQ
babyAFQ
AFQ
babyAFQ
AFQ
babyAFQ
AFQ
babyAFQ

Forceps Minor
Newborn

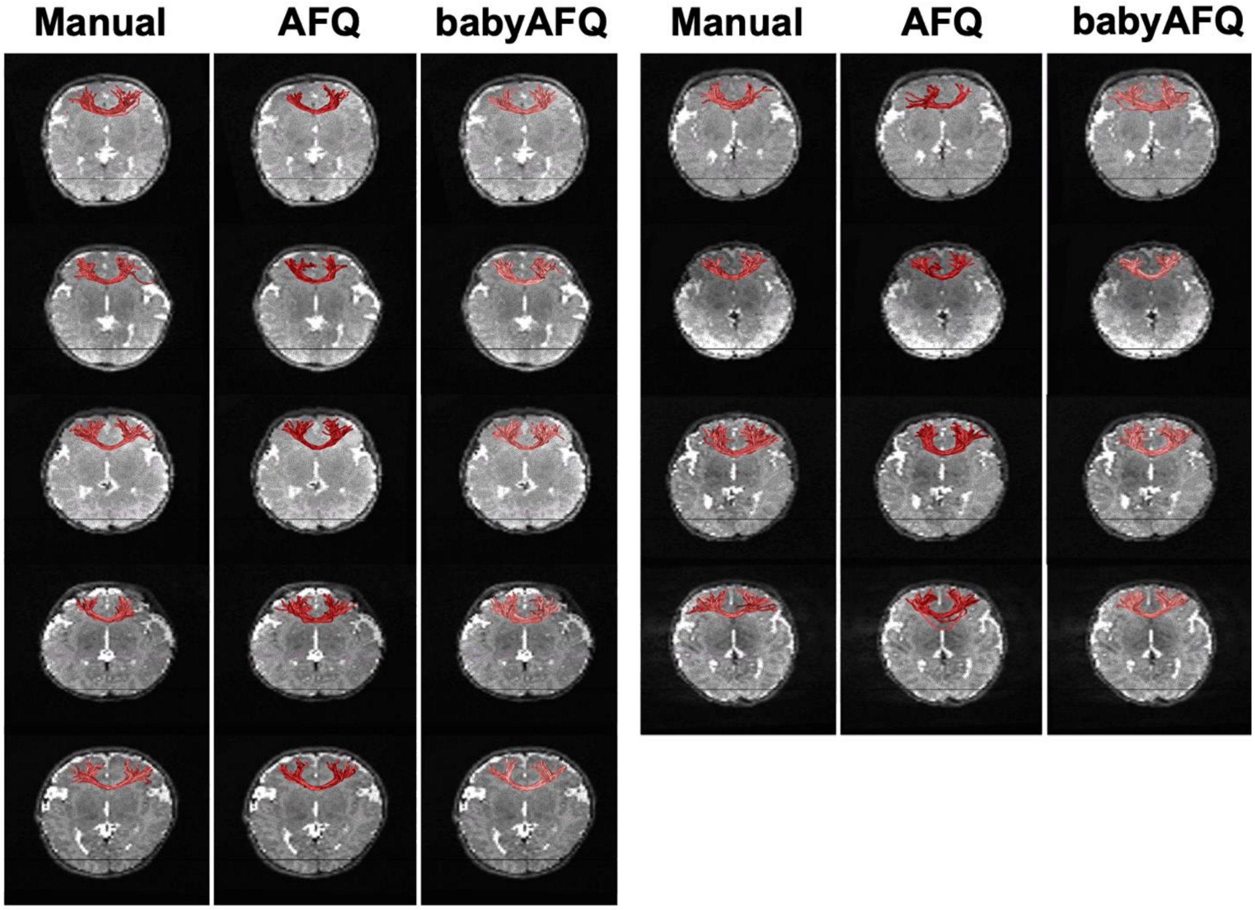

Forceps Minor

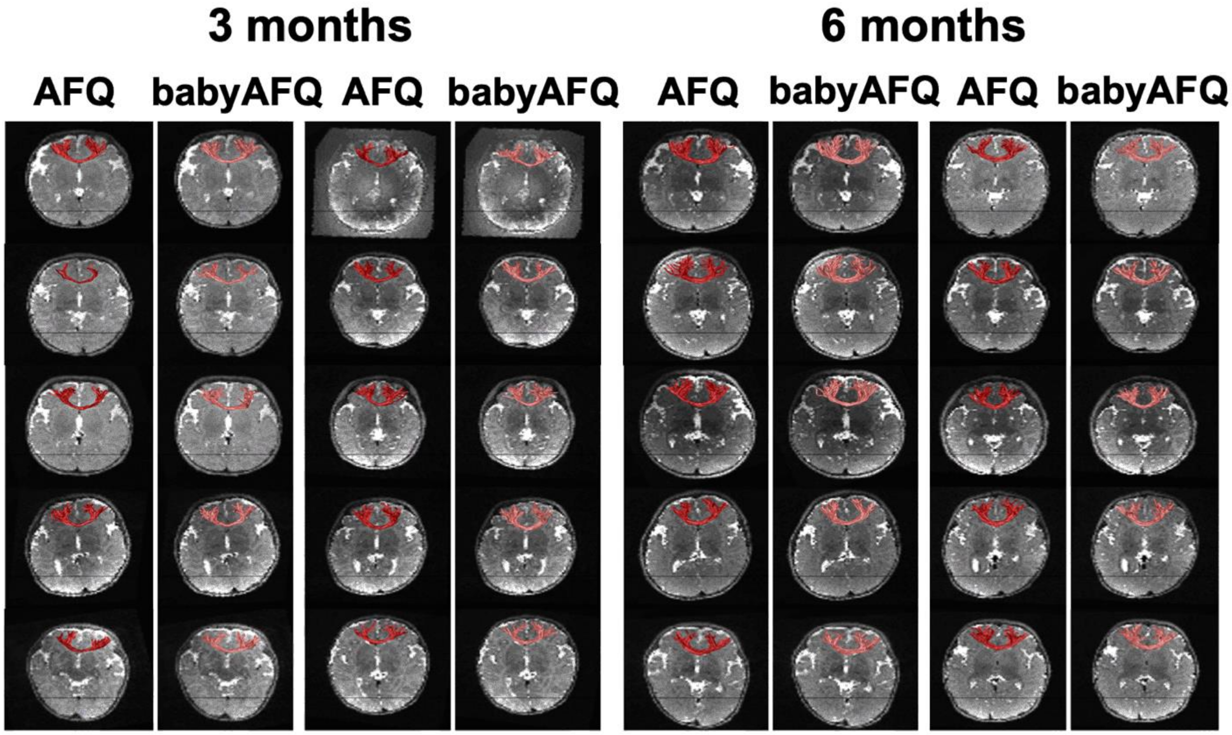

Left Inferior Frontal Occipital Fasciculus
Newborn

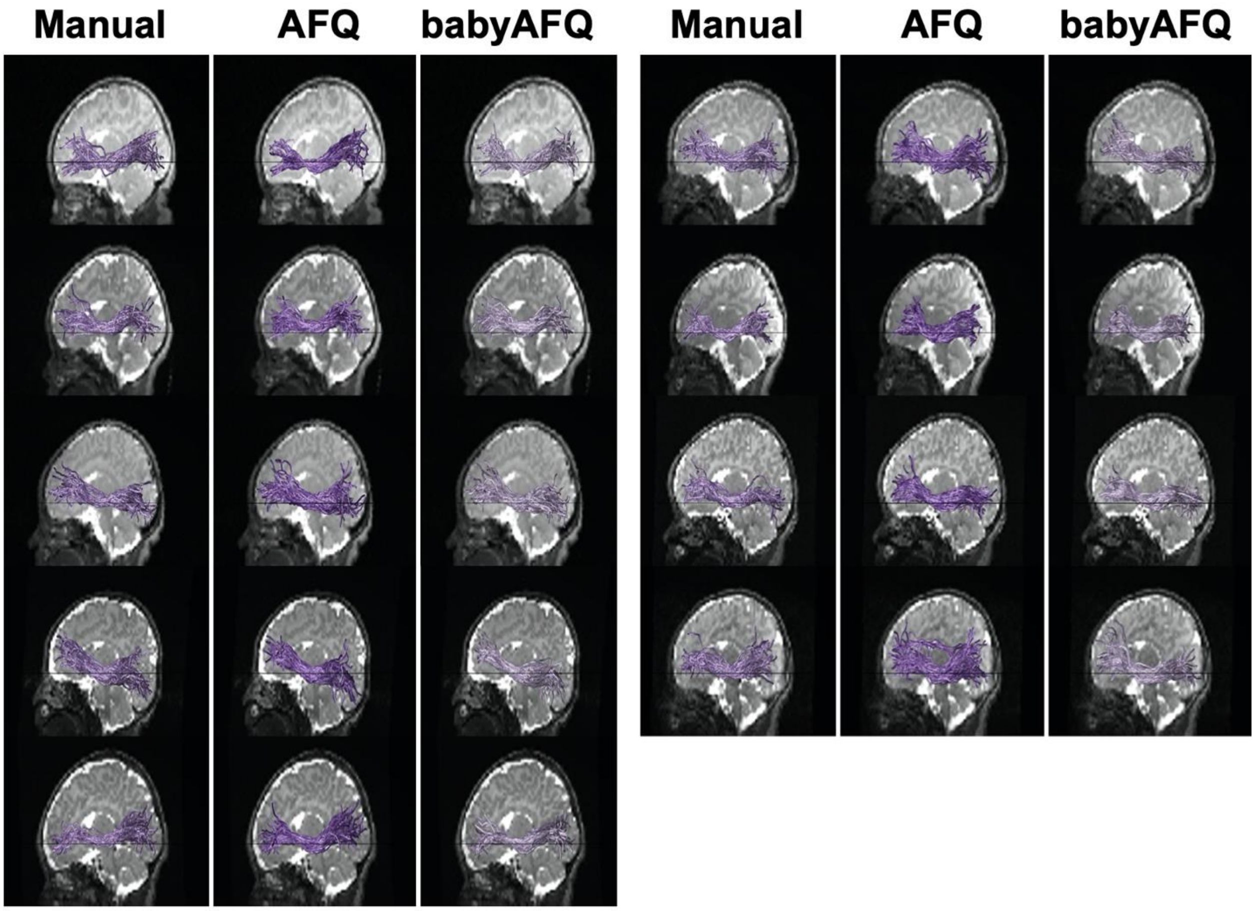

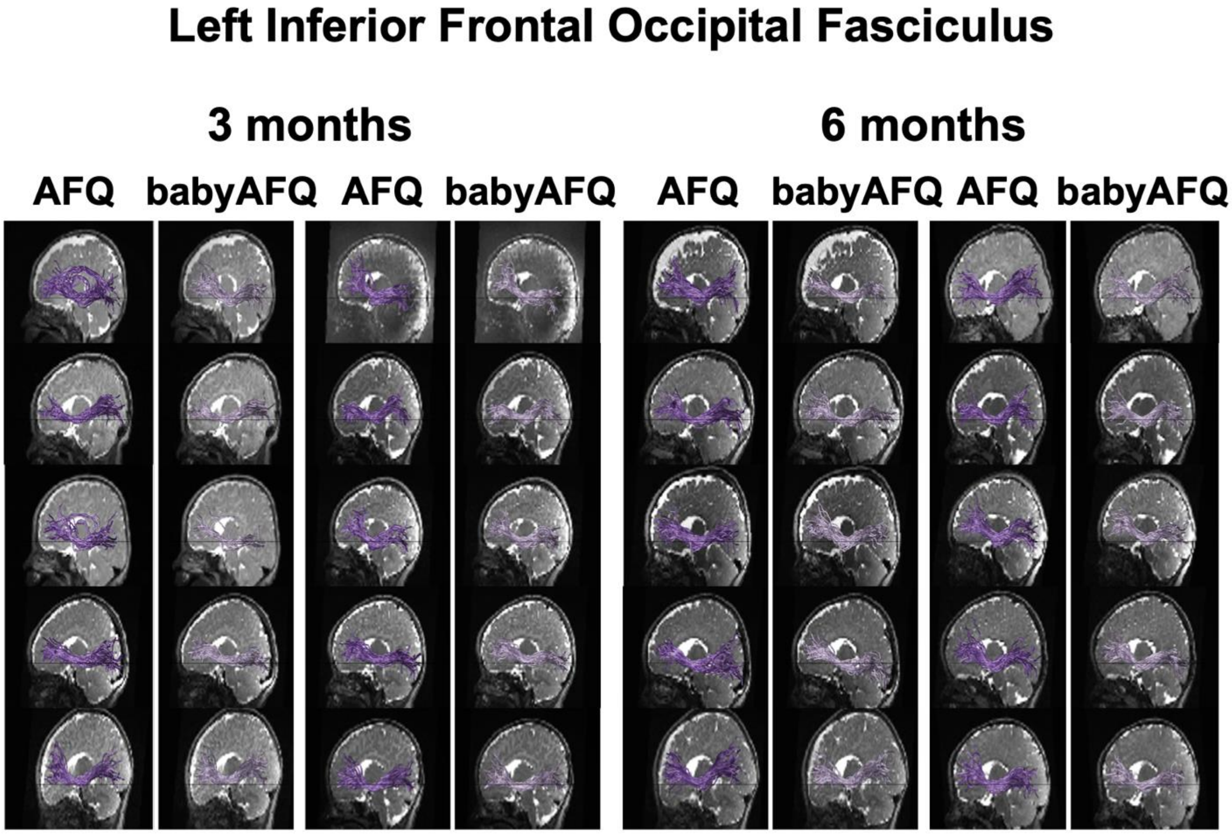
Left Inferior Frontal Occipital Fasciculus
3 months
6 months
AFQ
babyAFQ
AFQ
babyAFQ
AFQ
babyAFQ
AFQ
babyAFQ

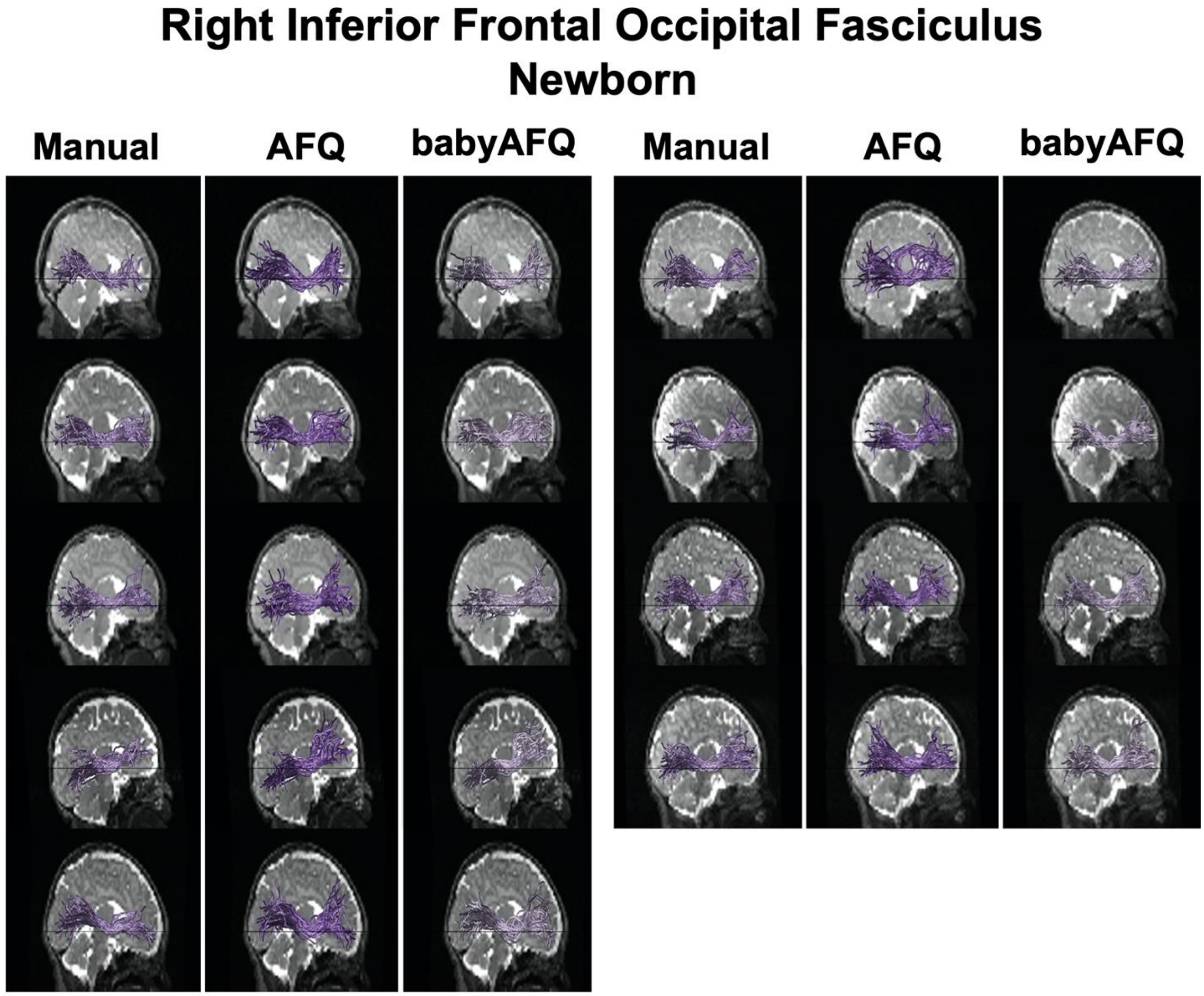
Right Inferior Frontal Occipital Fasciculus
Newborn
Manual
AFQ
babyAFQ
Manual
AFQ
babyAFQ

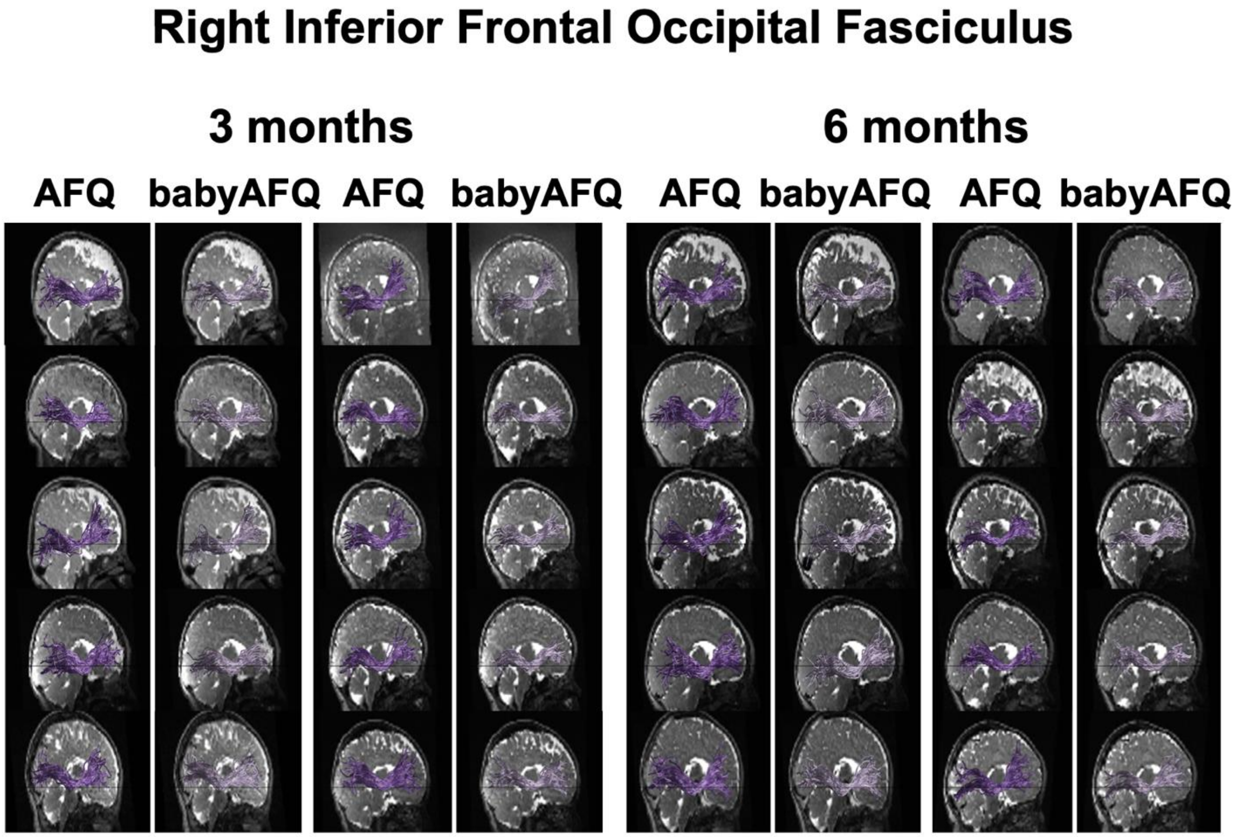
Right Inferior Frontal Occipital Fasciculus
3 months
6 months
AFQ
babyAFQ
AFQ
babyAFQ
AFQ
babyAFQ
AFQ
babyAFQ

Left Inferior Longitudinal Fasciculus
Newborn

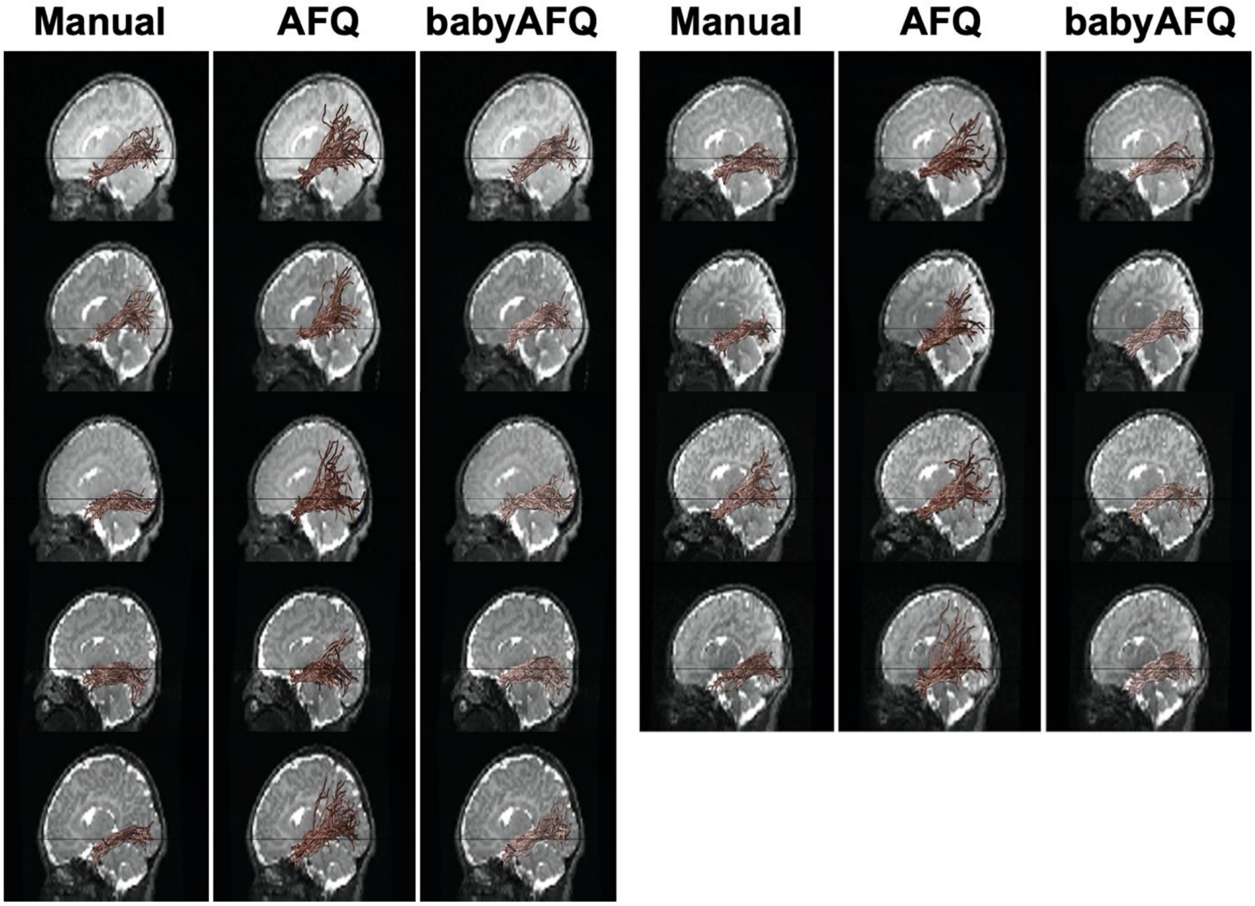

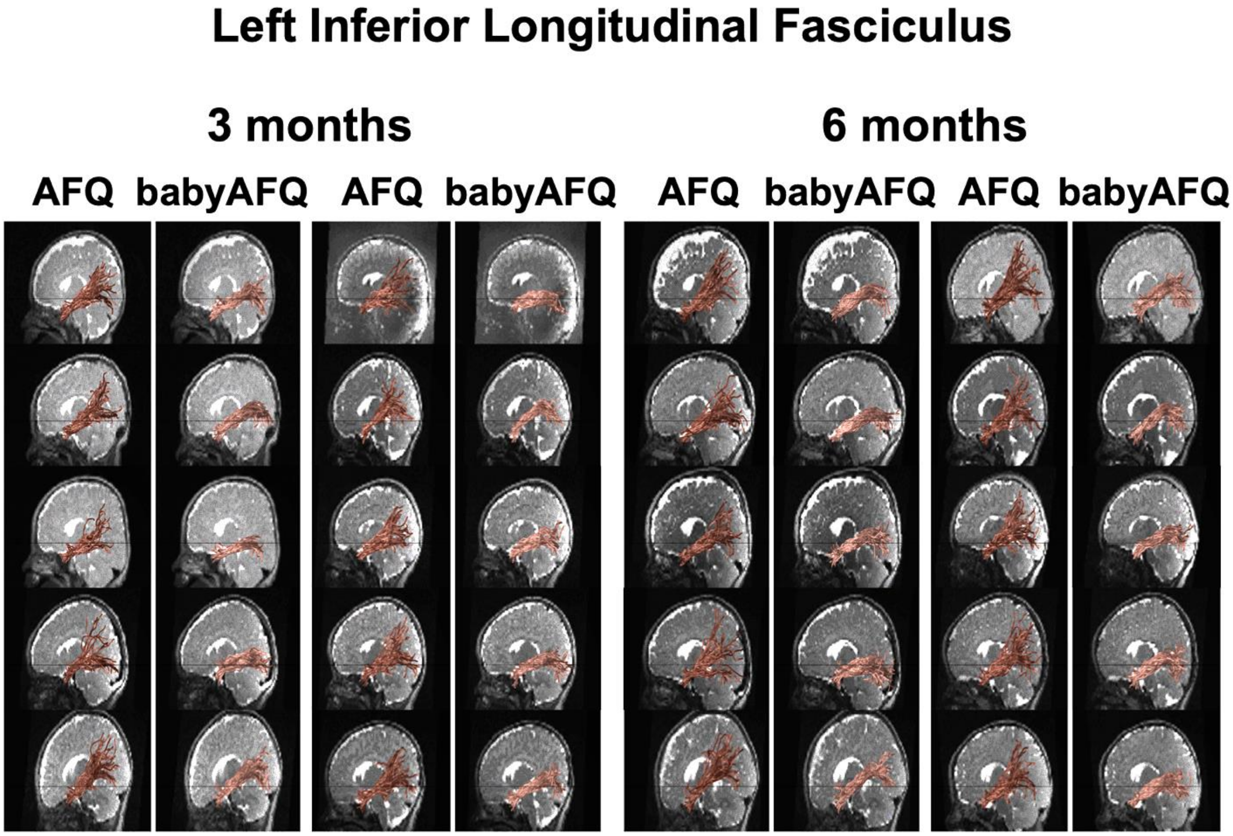
Left Inferior Longitudinal Fasciculus
3 months
6 months
AFQ
babyAFQ
AFQ
babyAFQ
AFQ
babyAFQ
AFQ
babyAFQ

Right Inferior Longitudinal Fasciculus
Newborn

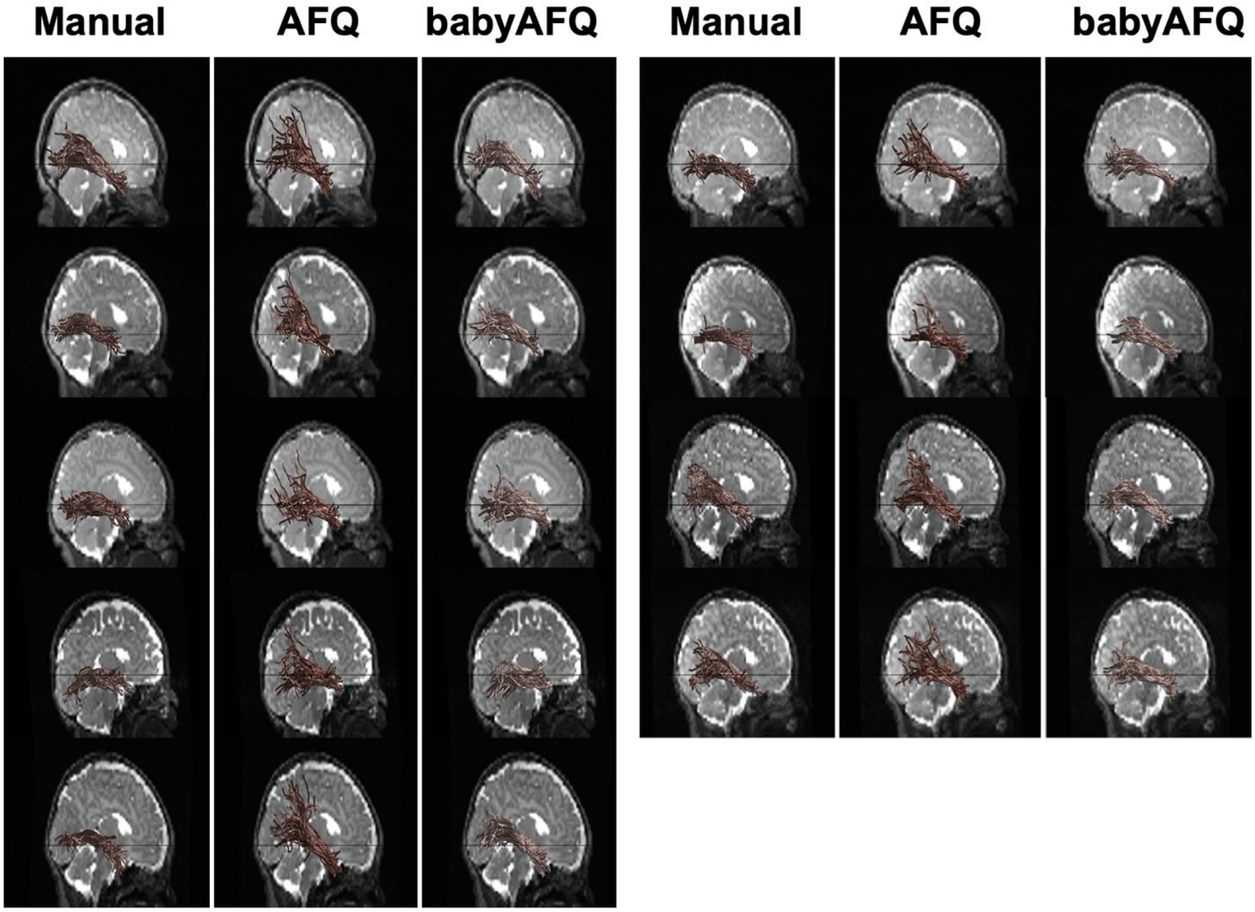

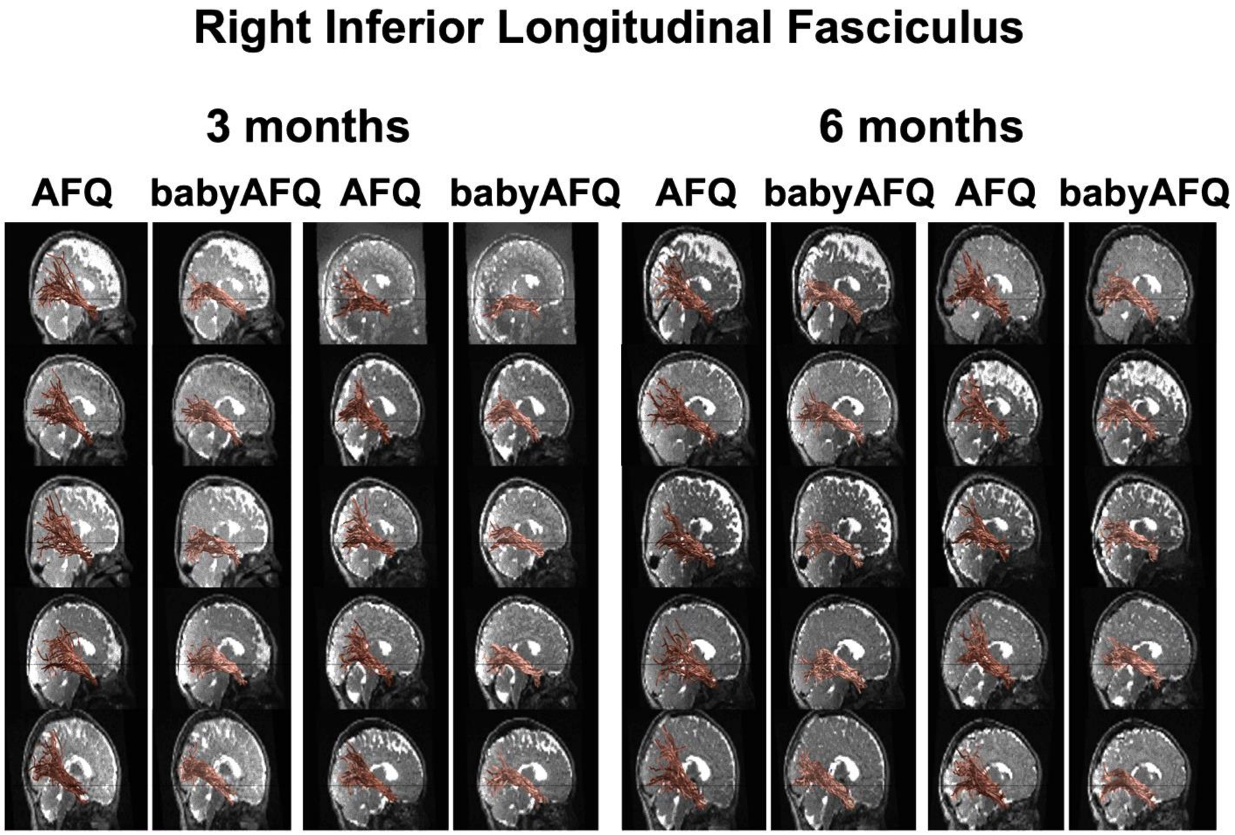
Right Inferior Longitudinal Fasciculus
3 months
6 months
AFQ
babyAFQ
AFQ
babyAFQ
AFQ
babyAFQ
AFQ
babyAFQ

Left Middle Longitudinal Fasciculus

Newborn | 3 months | 6 months

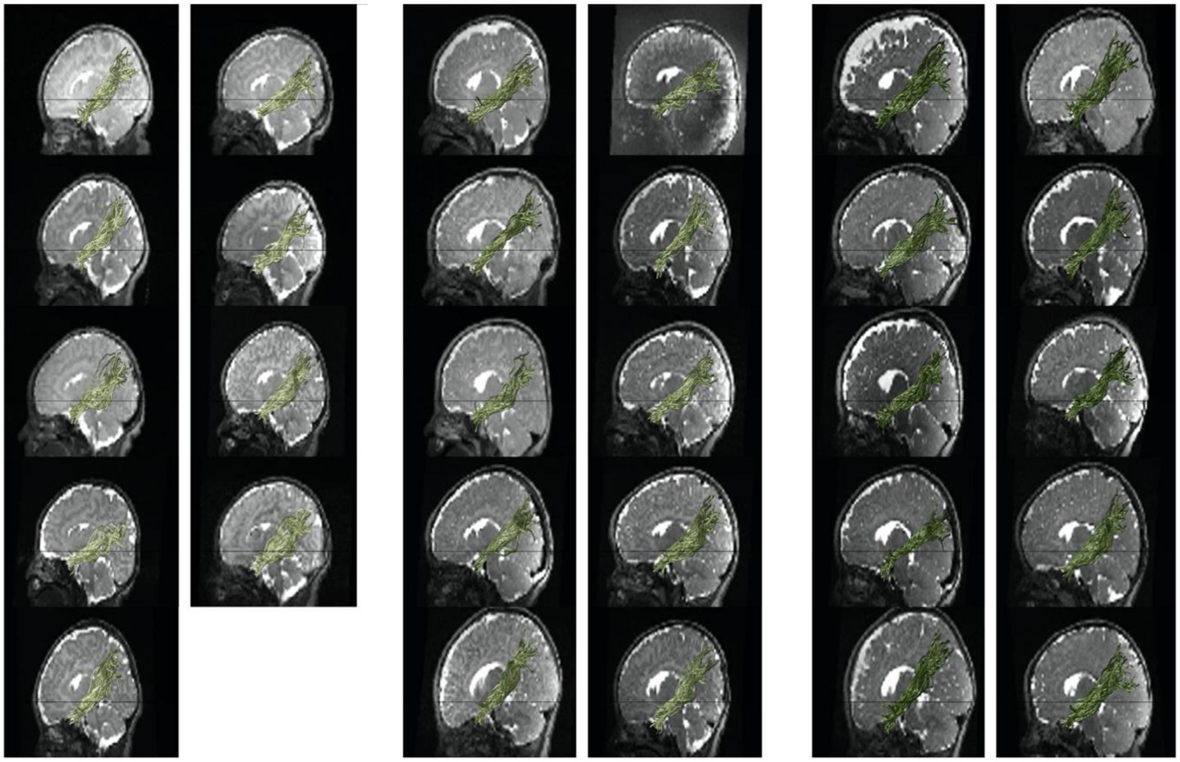

Right Middle Longitudinal Fasciculus

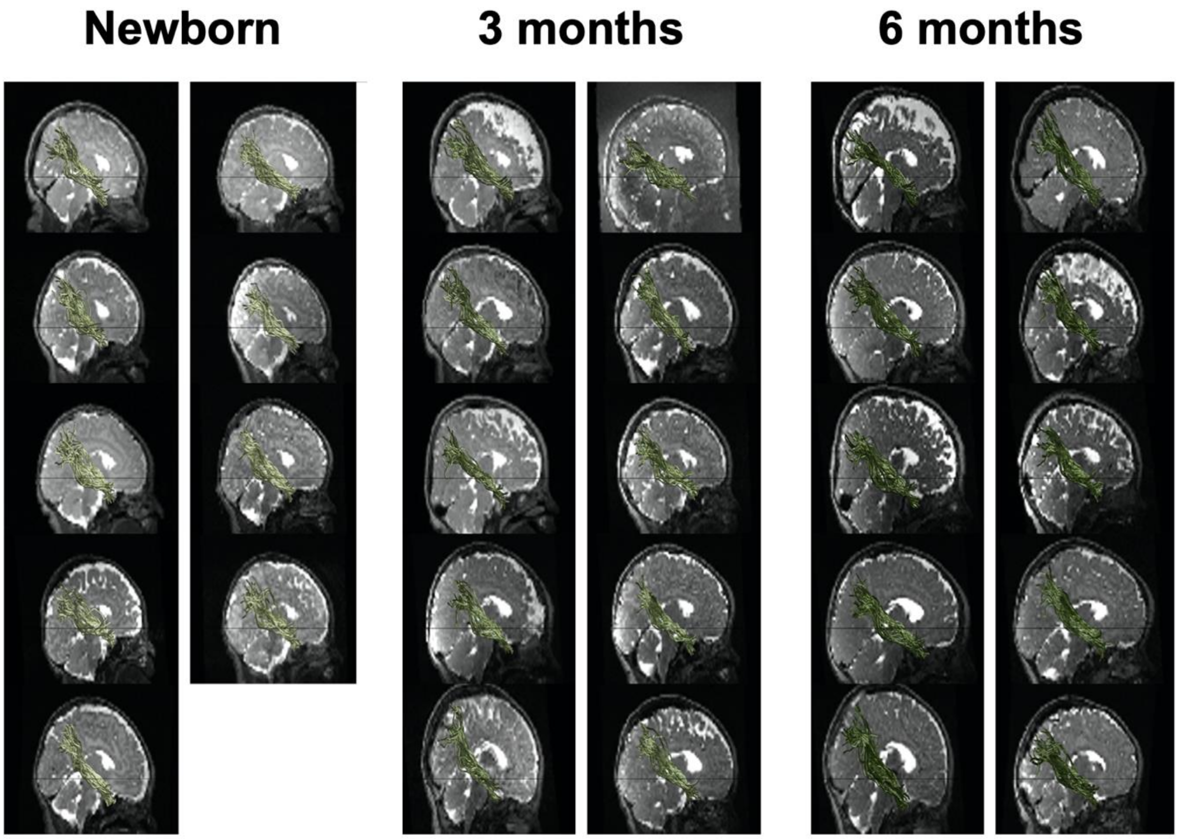

Left Superior Longitudinal Fasciculus
Newborn

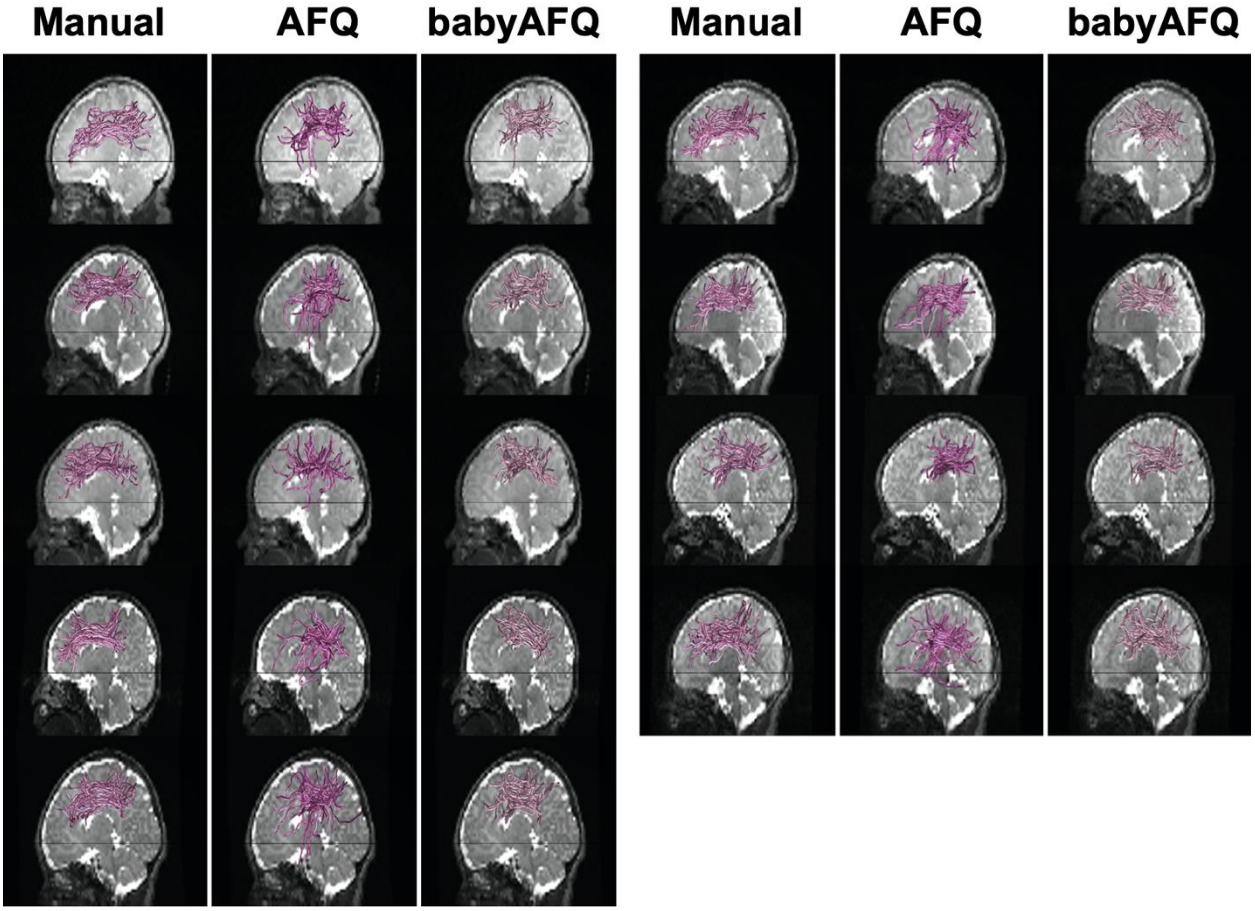

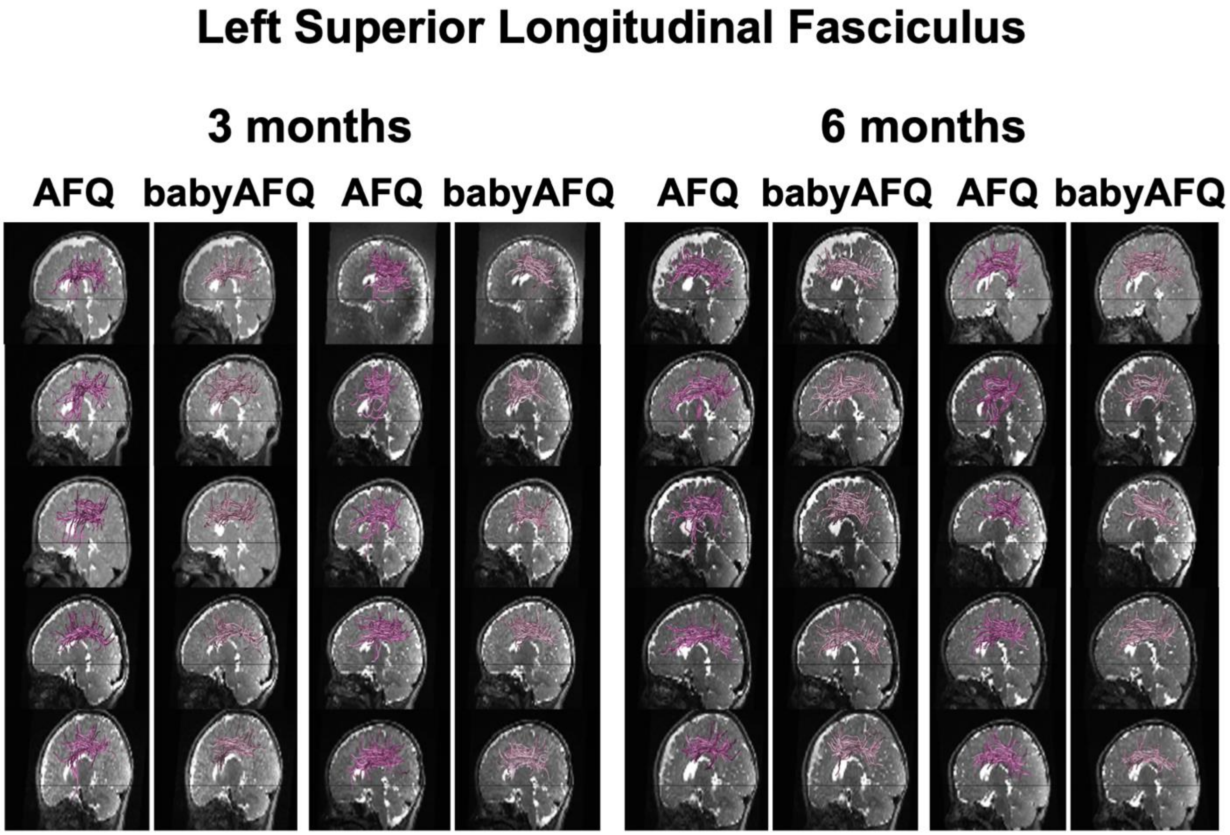
Left Superior Longitudinal Fasciculus
3 months
6 months
AFQ
babyAFQ
AFQ
babyAFQ
AFQ
babyAFQ
AFQ
babyAFQ

Right Superior Longitudinal Fasciculus
Newborn

Manual AFQ babyAFQ Manual AFQ babyAFQ

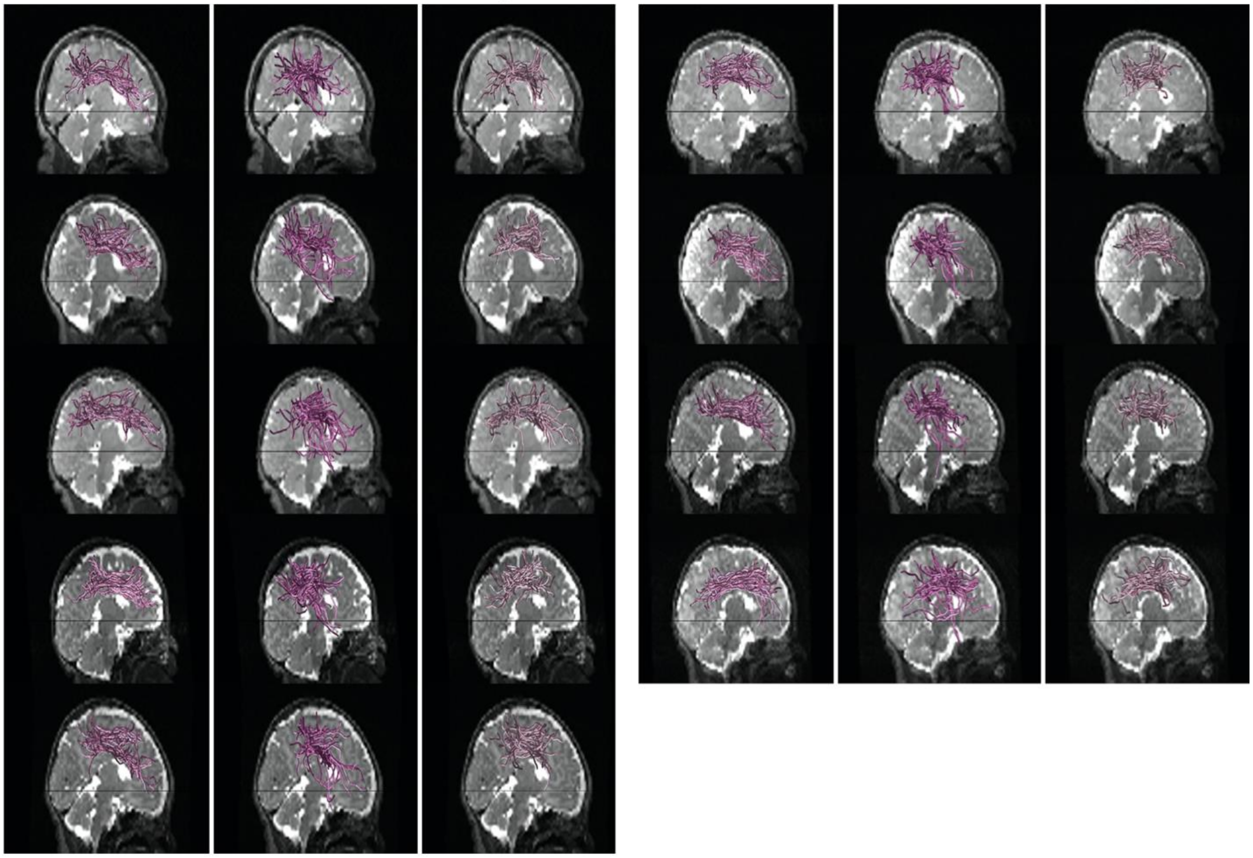

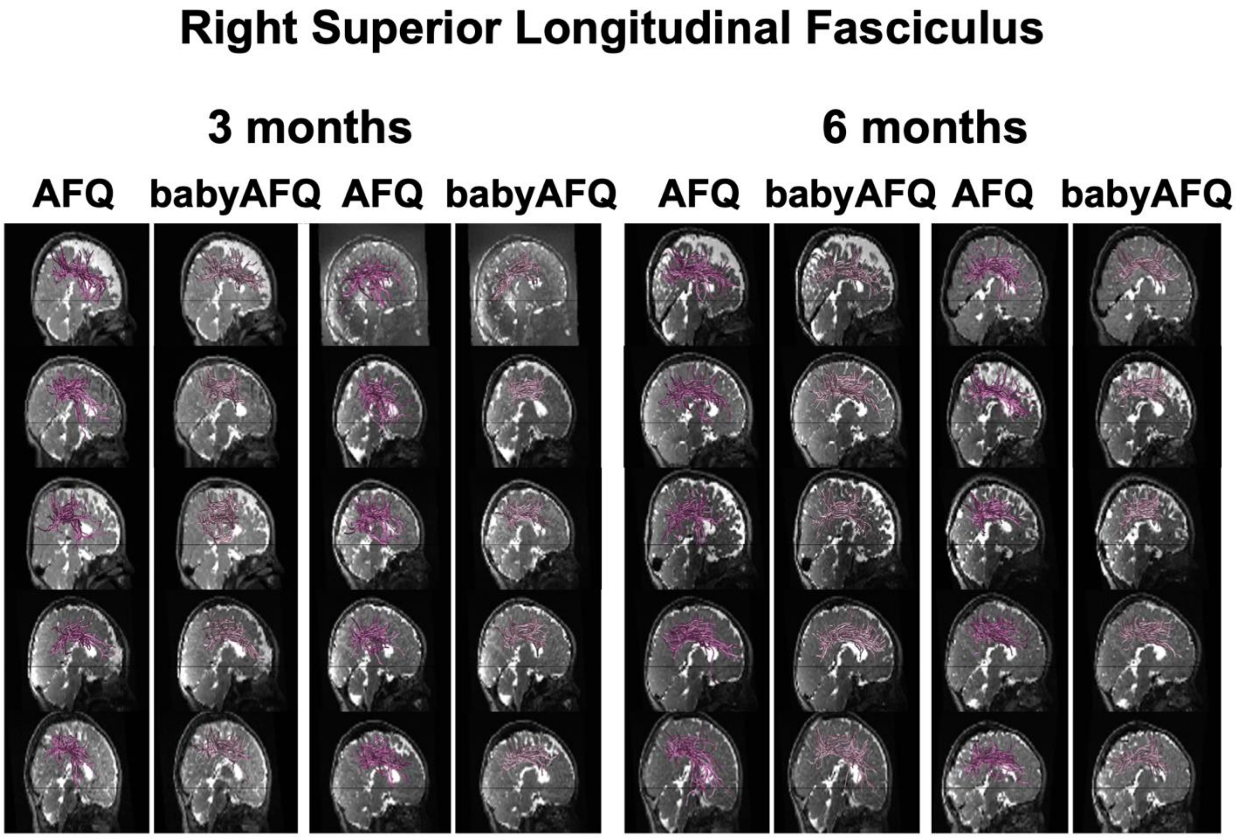
Right Superior Longitudinal Fasciculus
3 months
6 months
AFQ
babyAFQ
AFQ
babyAFQ
AFQ
babyAFQ
AFQ
babyAFQ

Left Uncinate Fasciculus
Newborn

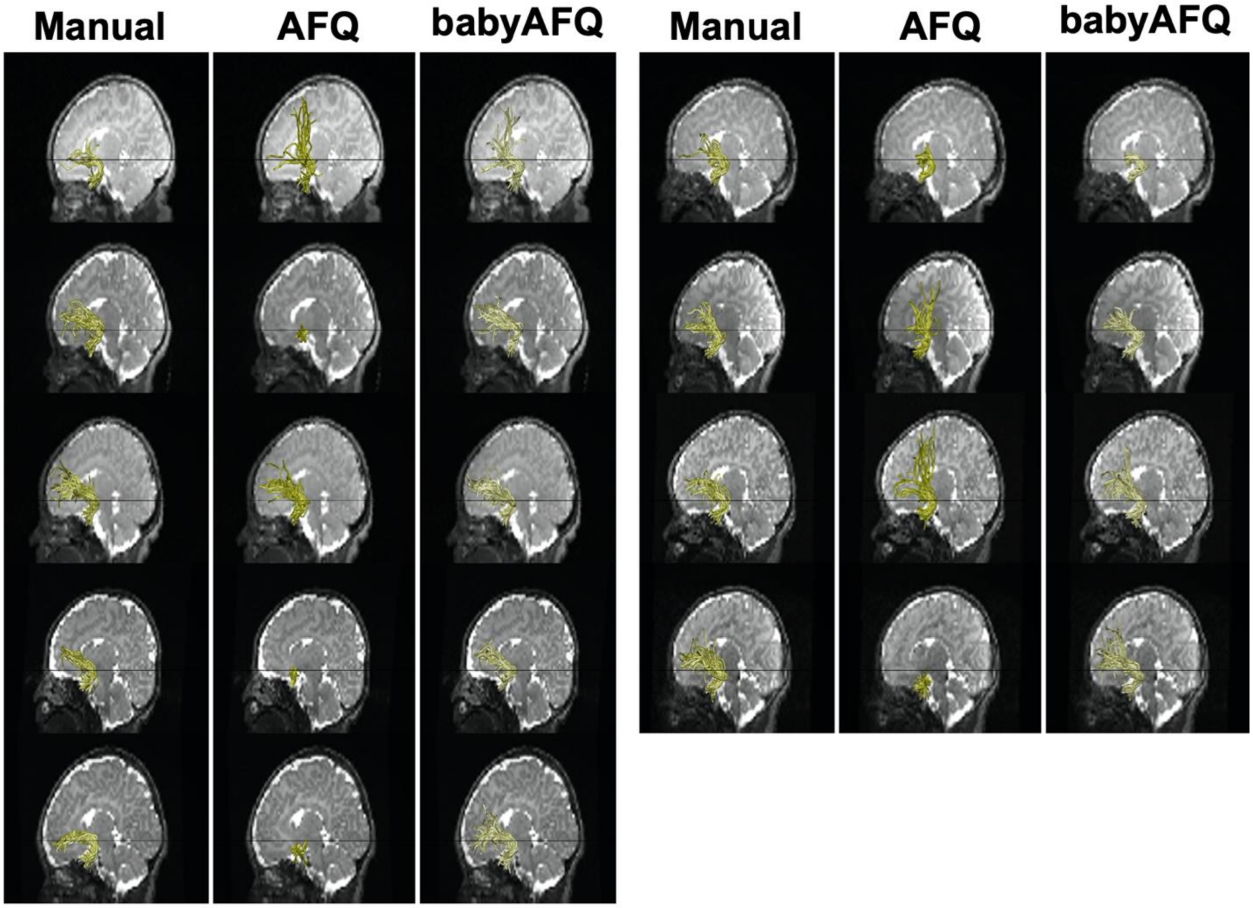

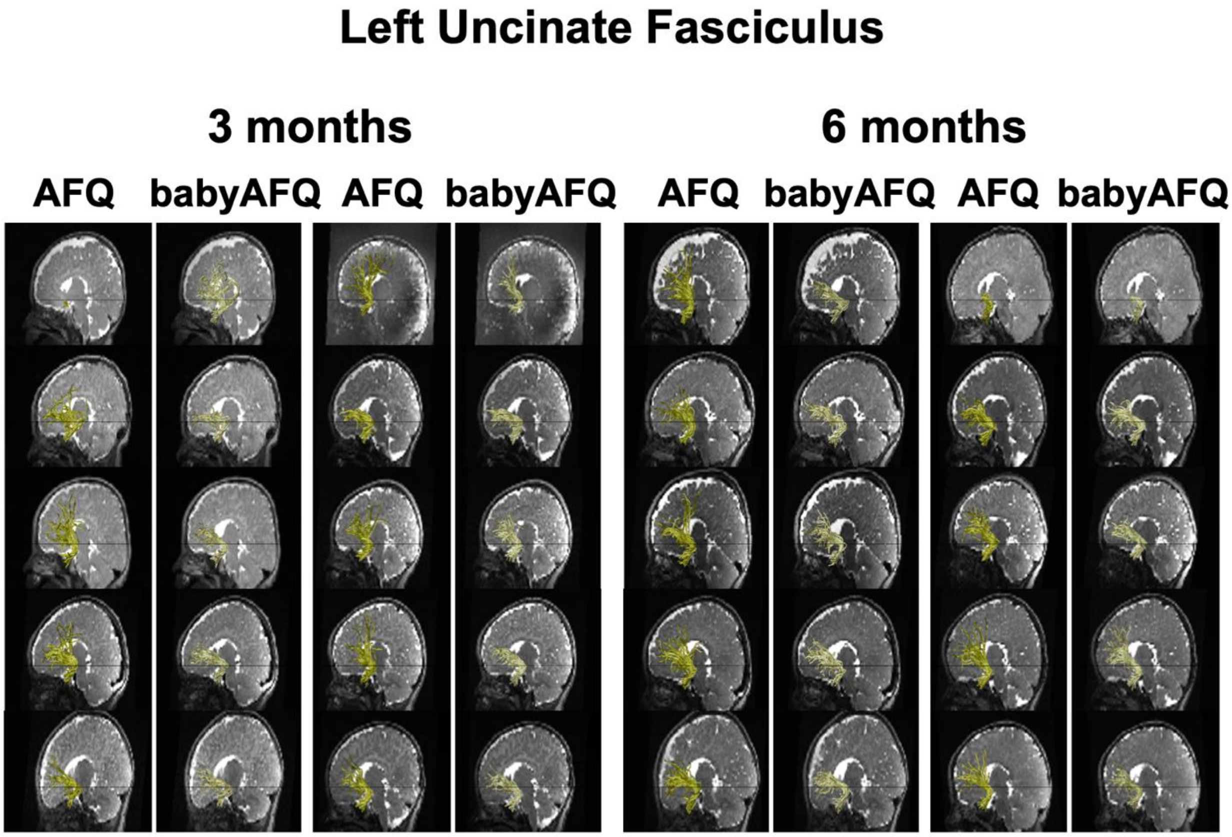
Left Uncinate Fasciculus
3 months
6 months
AFQ
babyAFQ
AFQ
babyAFQ
AFQ
babyAFQ
AFQ
babyAFQ

Right Uncinate Fasciculus
Newborn

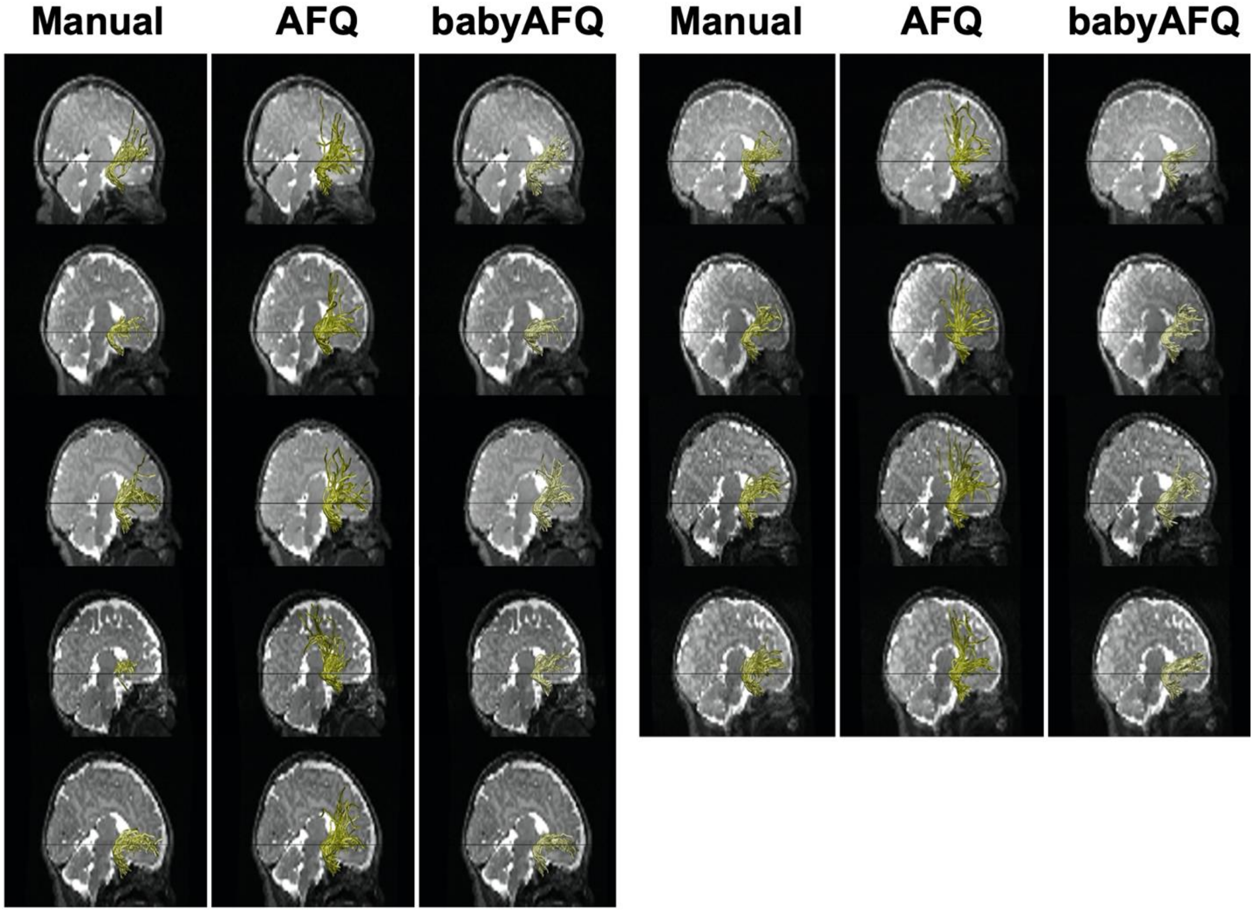

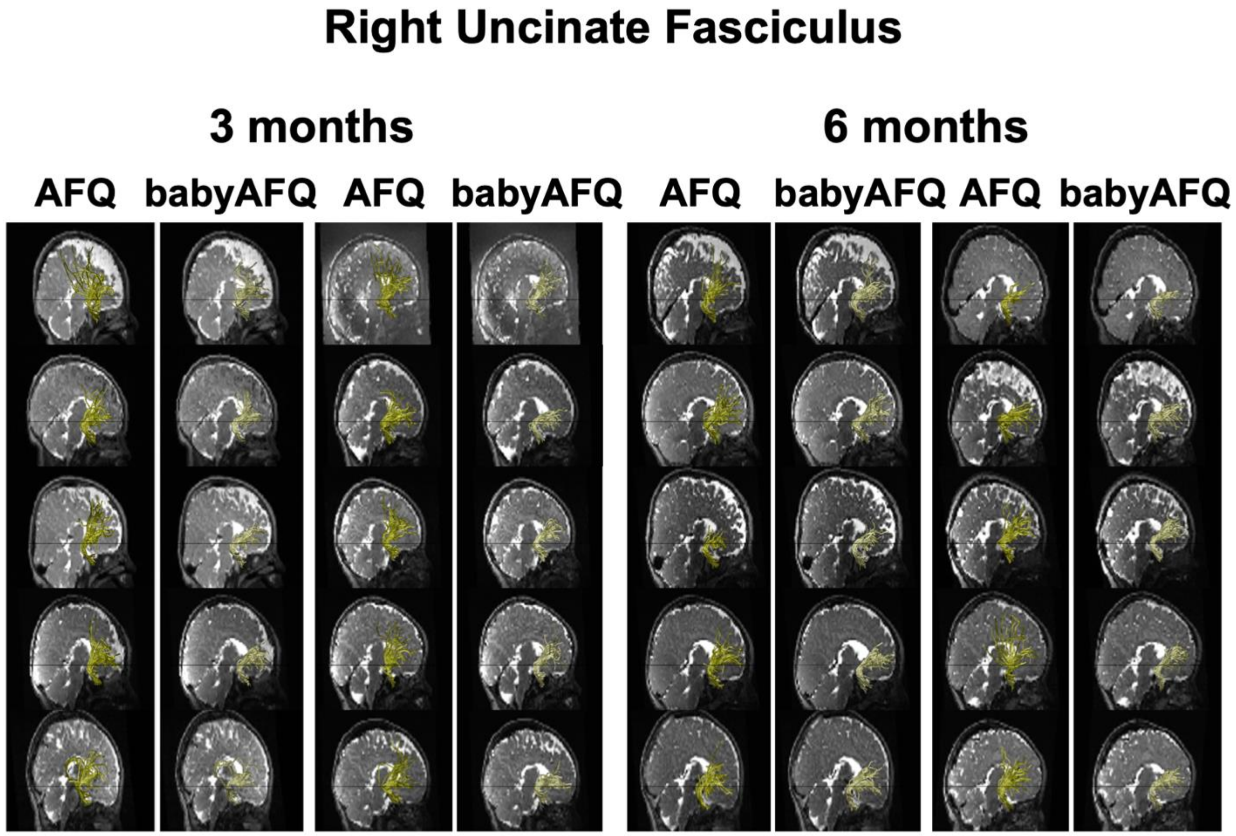
Right Uncinate Fasciculus
3 months
6 months
AFQ
babyAFQ
AFQ
babyAFQ
AFQ
babyAFQ
AFQ
babyAFQ

Left Arcuate Fasciculus
Newborn

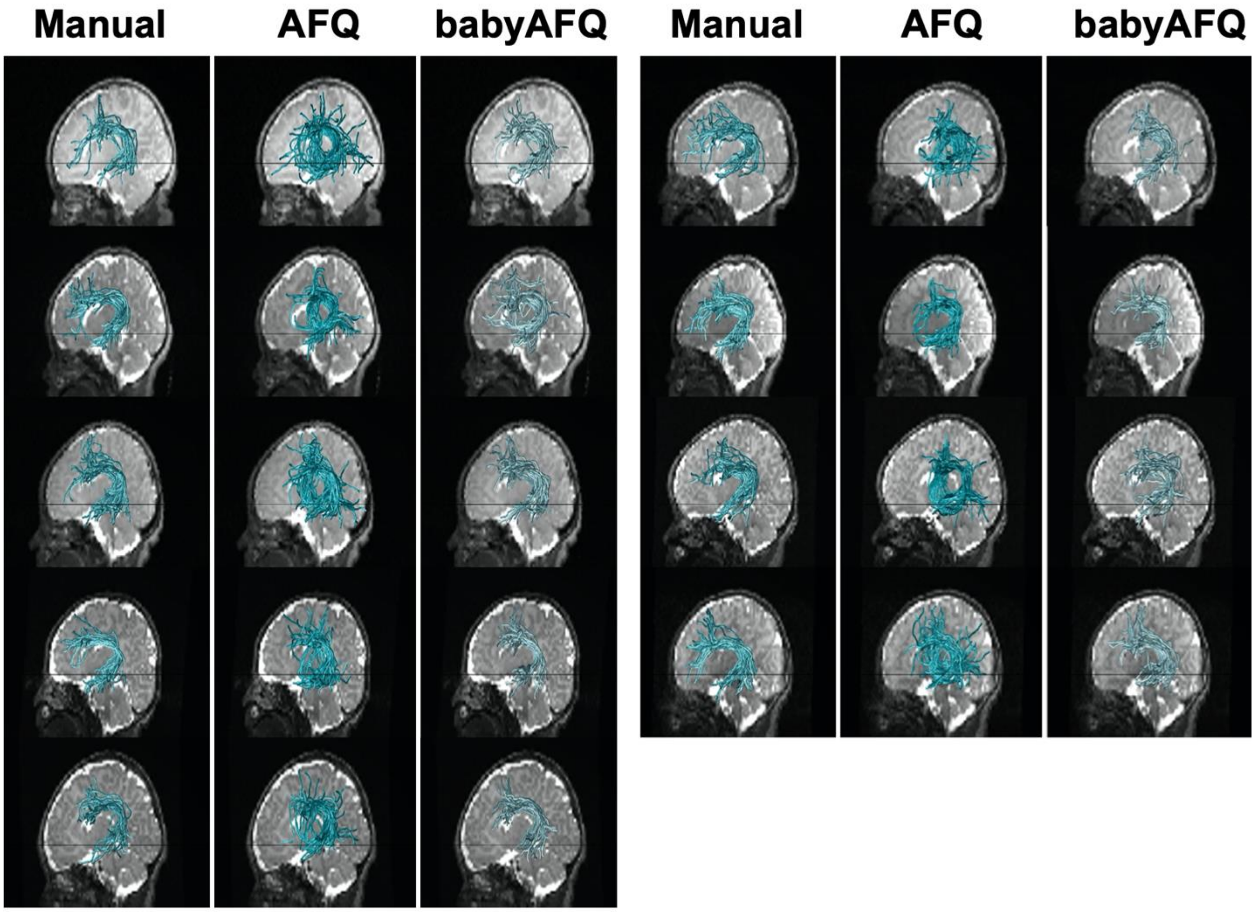

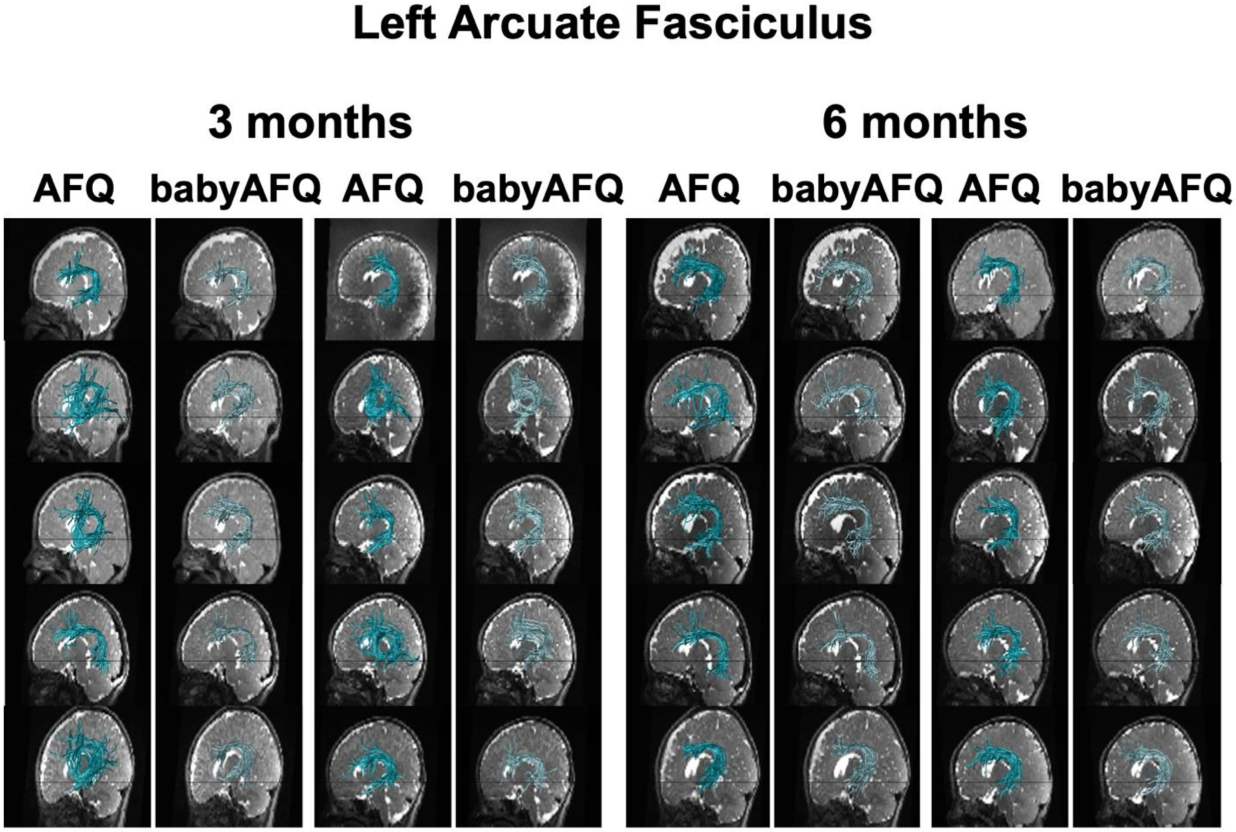
Left Arcuate Fasciculus
3 months
6 months
AFQ
babyAFQ
AFQ
babyAFQ
AFQ
babyAFQ
AFQ
babyAFQ

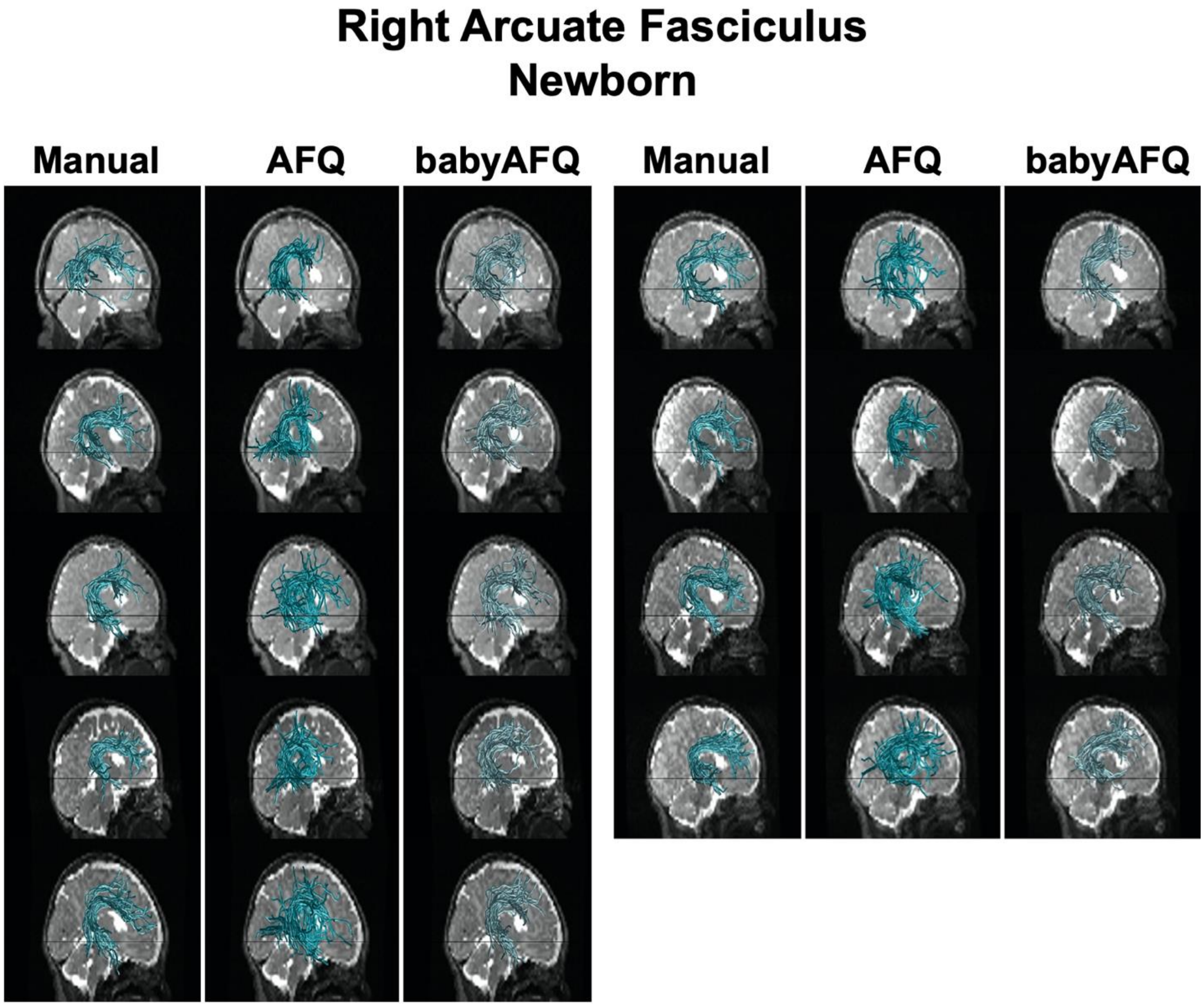
Right Arcuate Fasciculus
Newborn
Manual
AFQ
babyAFQ
Manual
AFQ
babyAFQ

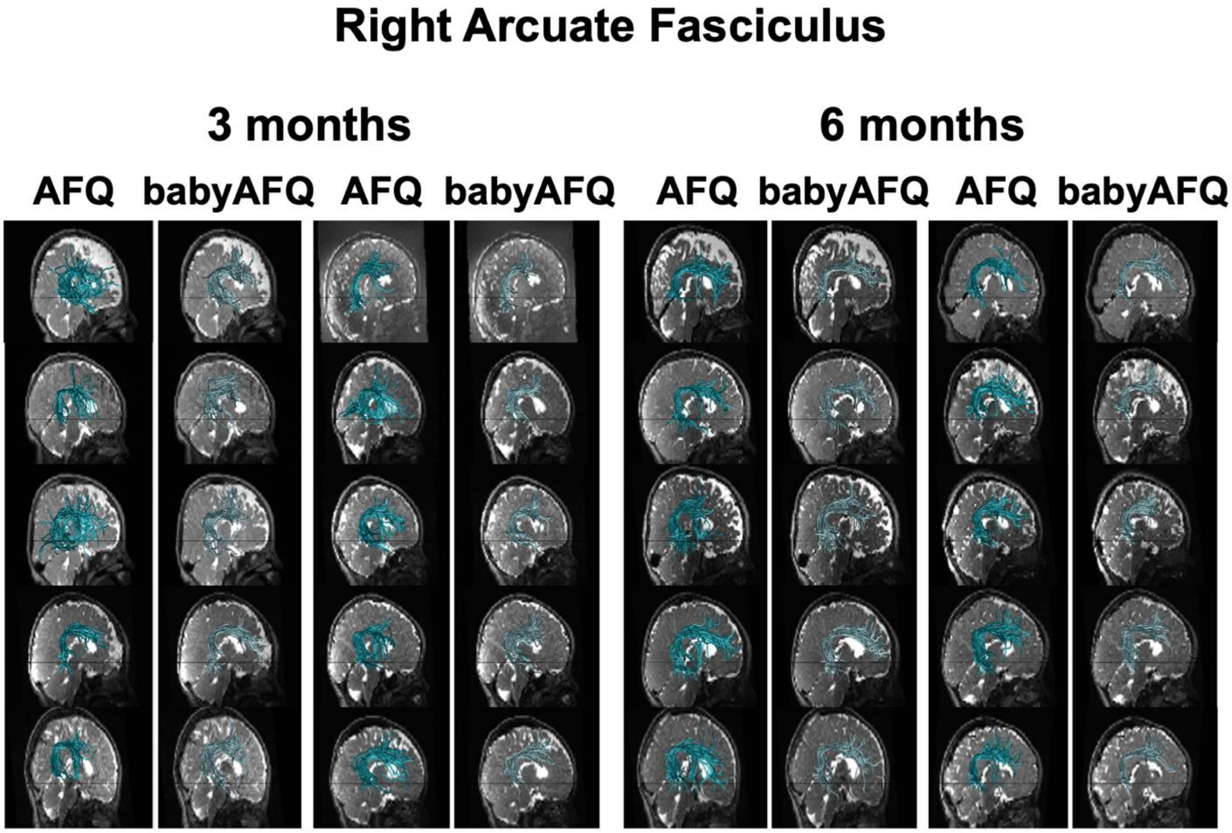
Right Arcuate Fasciculus
3 months
6 months
AFQ
babyAFQ
AFQ
babyAFQ
AFQ
babyAFQ
AFQ
babyAFQ

Left Posterior Arcuate Fasciculus
Newborn

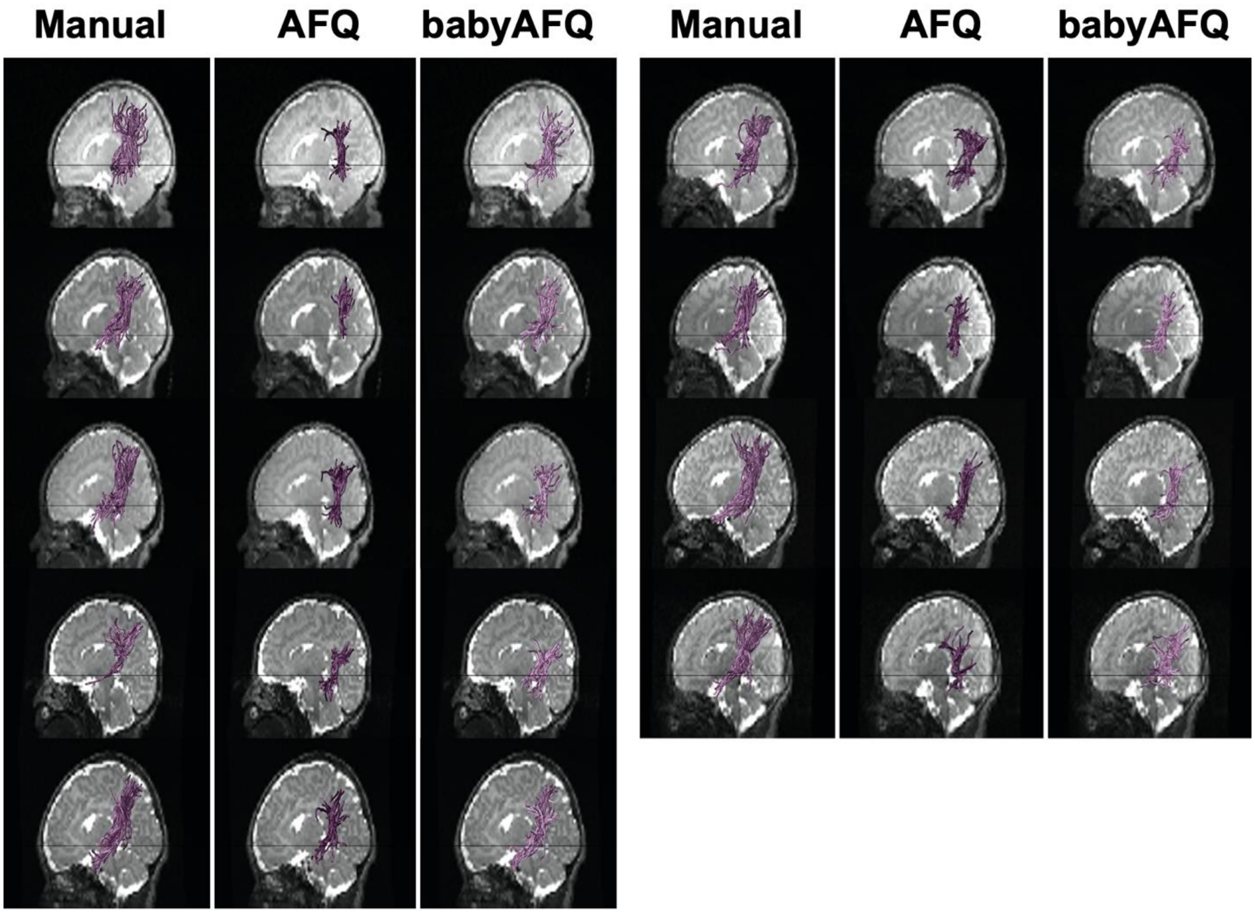

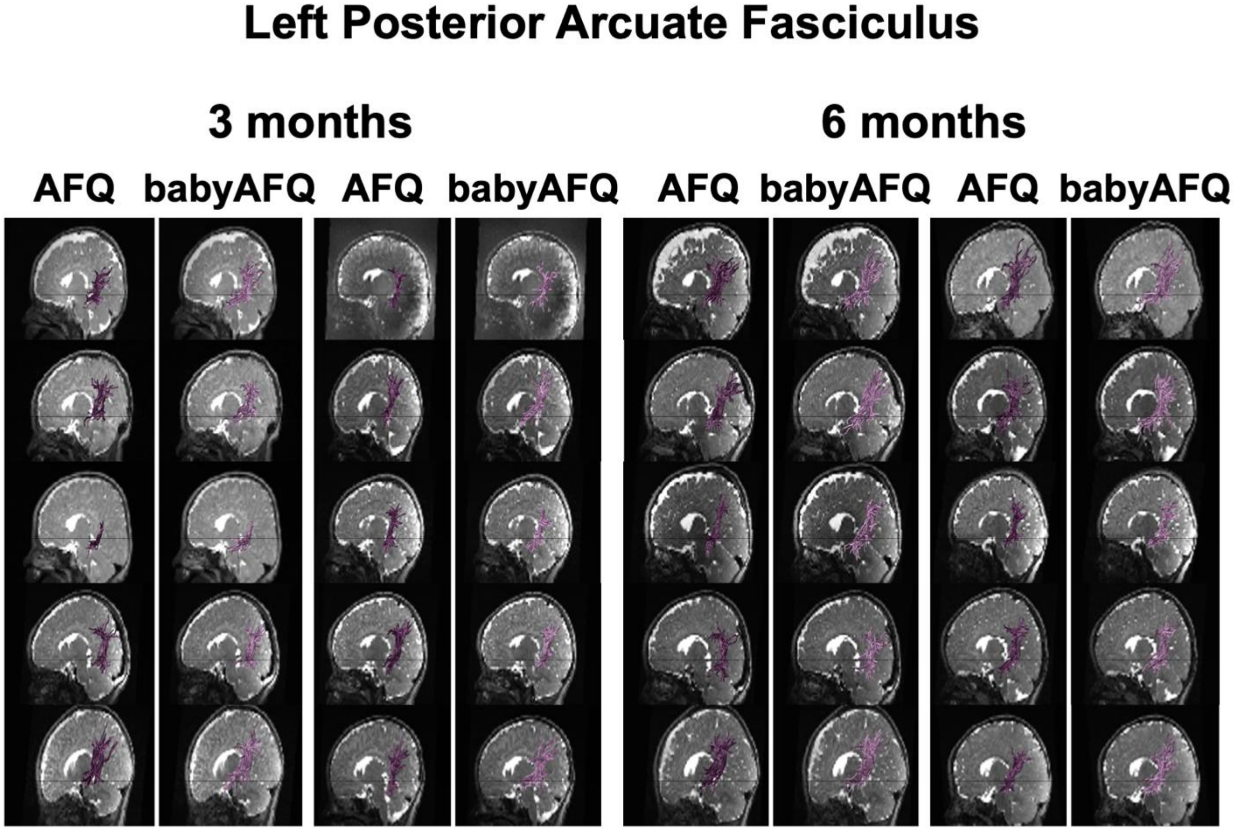
Left Posterior Arcuate Fasciculus
3 months
6 months
AFQ
babyAFQ
AFQ
babyAFQ
AFQ
babyAFQ
AFQ
babyAFQ

Right Posterior Arcuate Fasciculus
Newborn

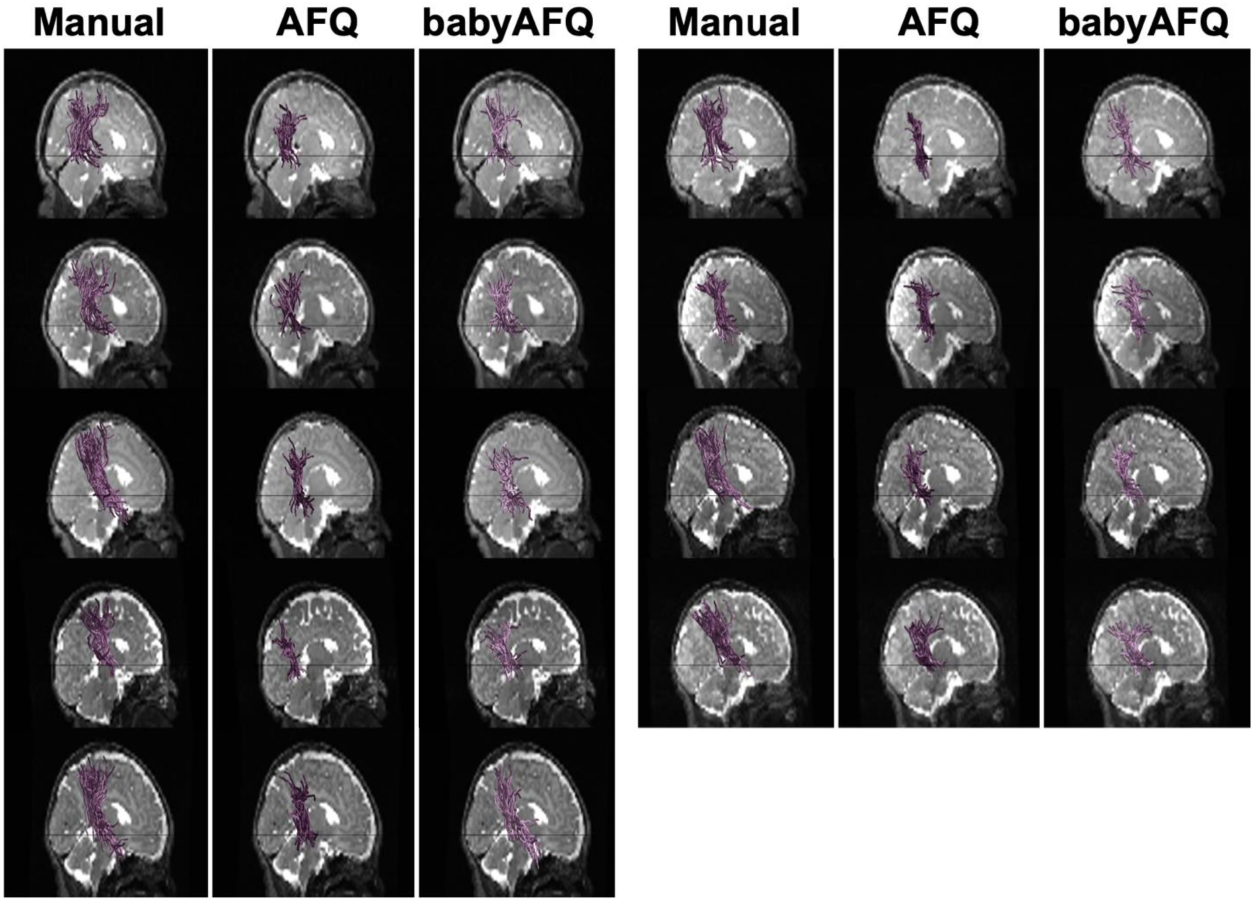

Right Posterior Arcuate Fasciculus

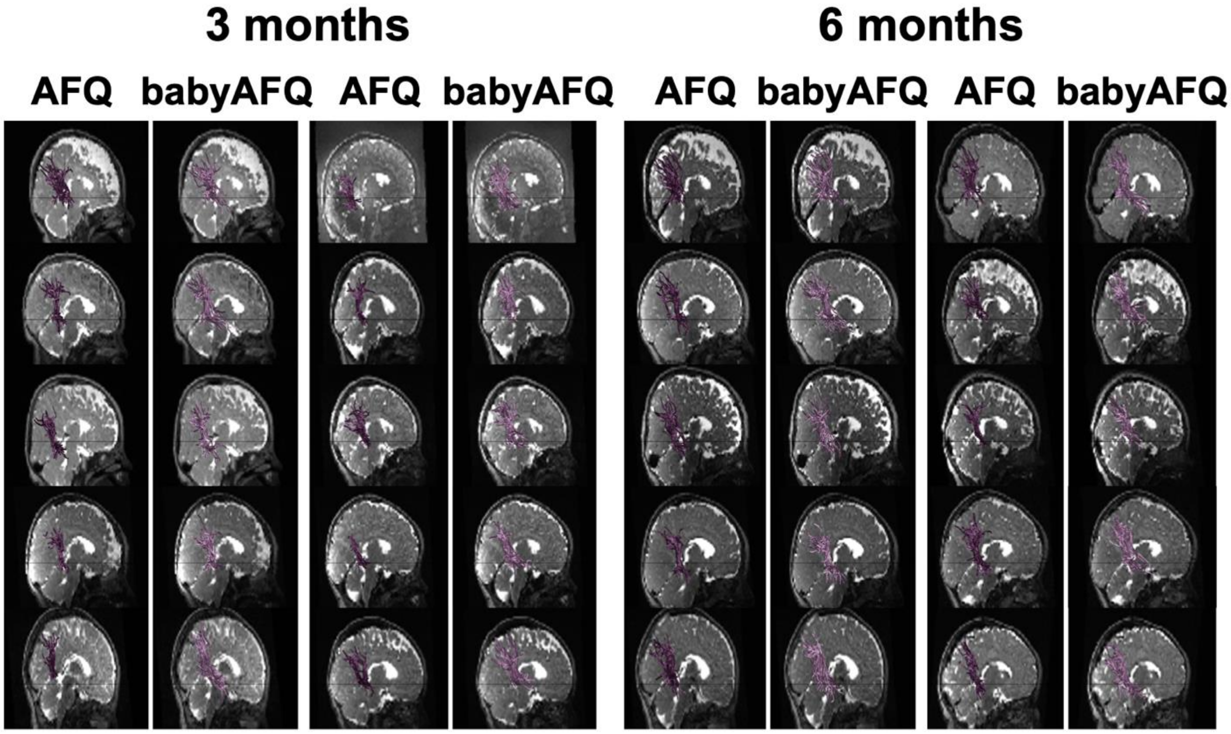

Left Vertical Occipital Fasciculus
Newborn

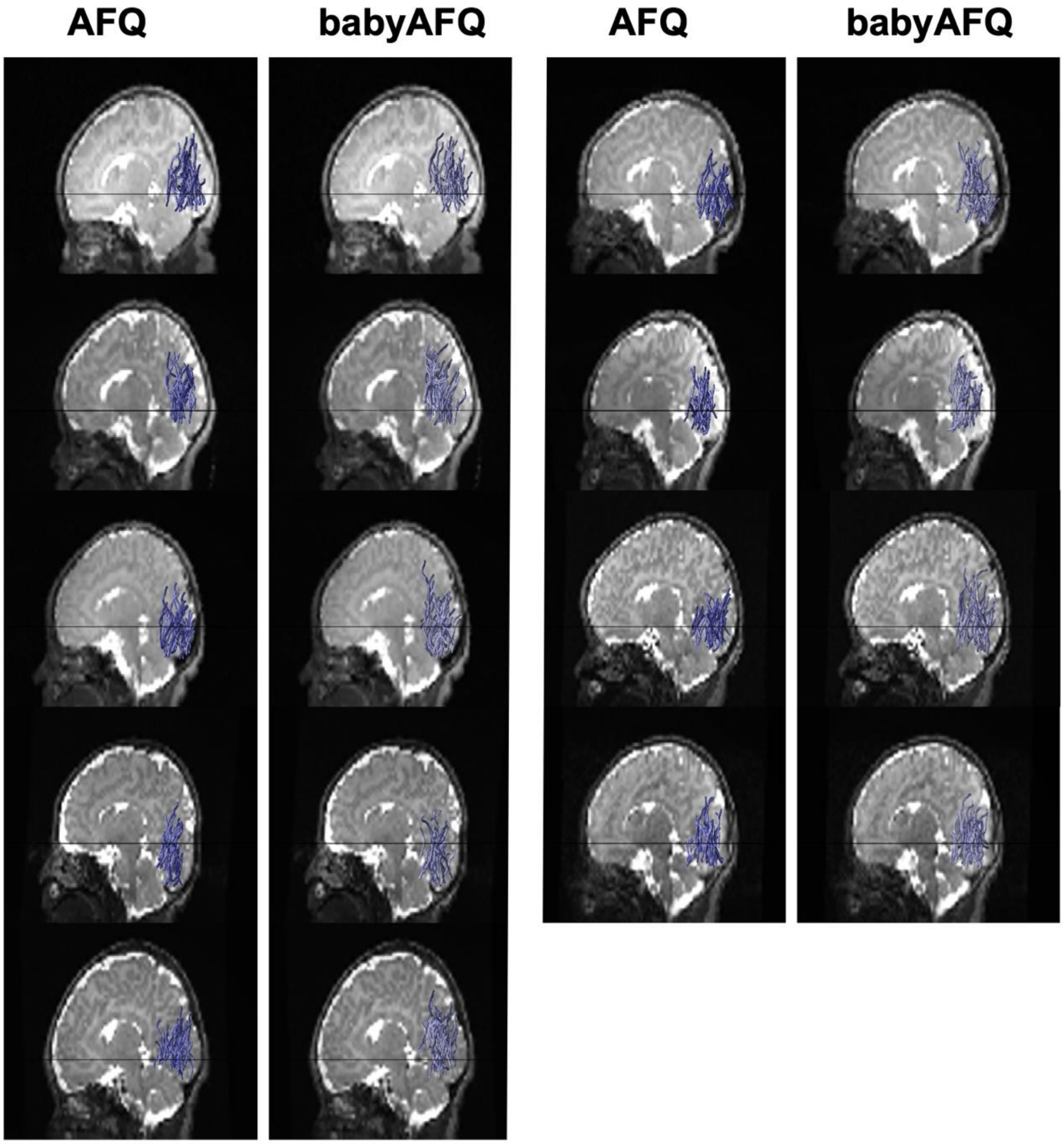

Left Vertical Occipital Fasciculus

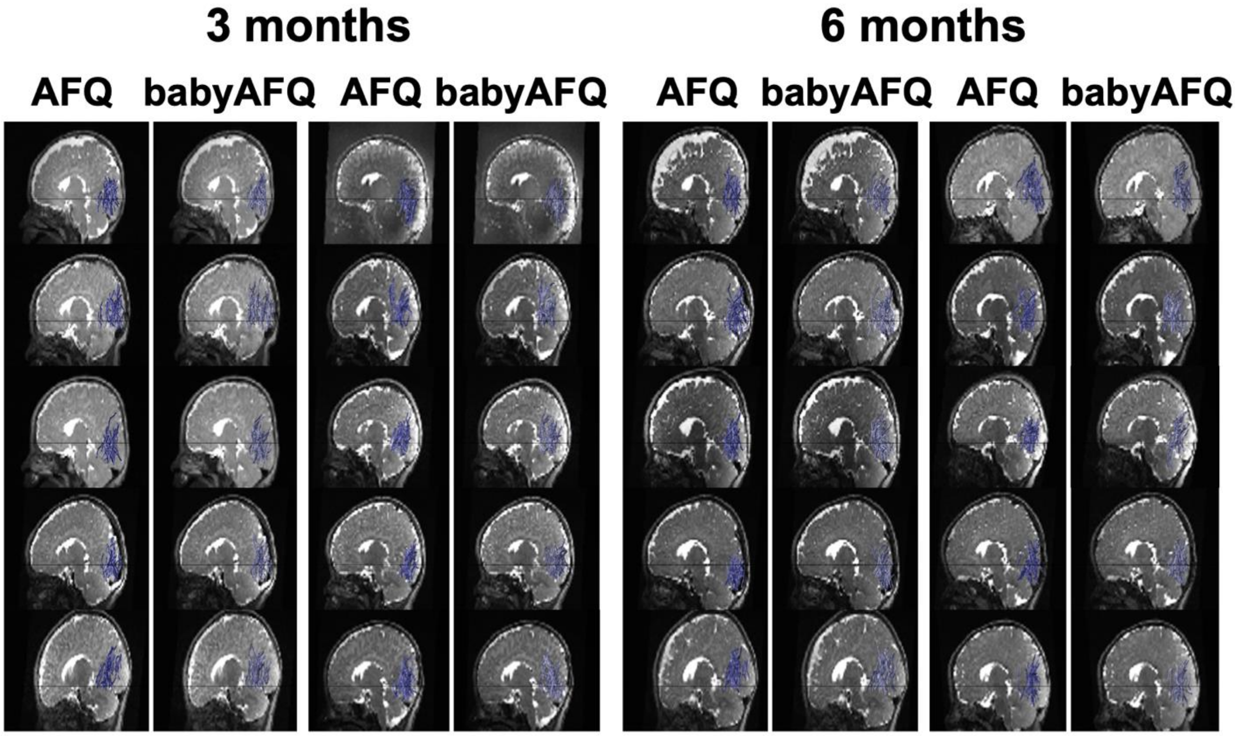

Right Vertical Occipital Fasciculus
Newborn

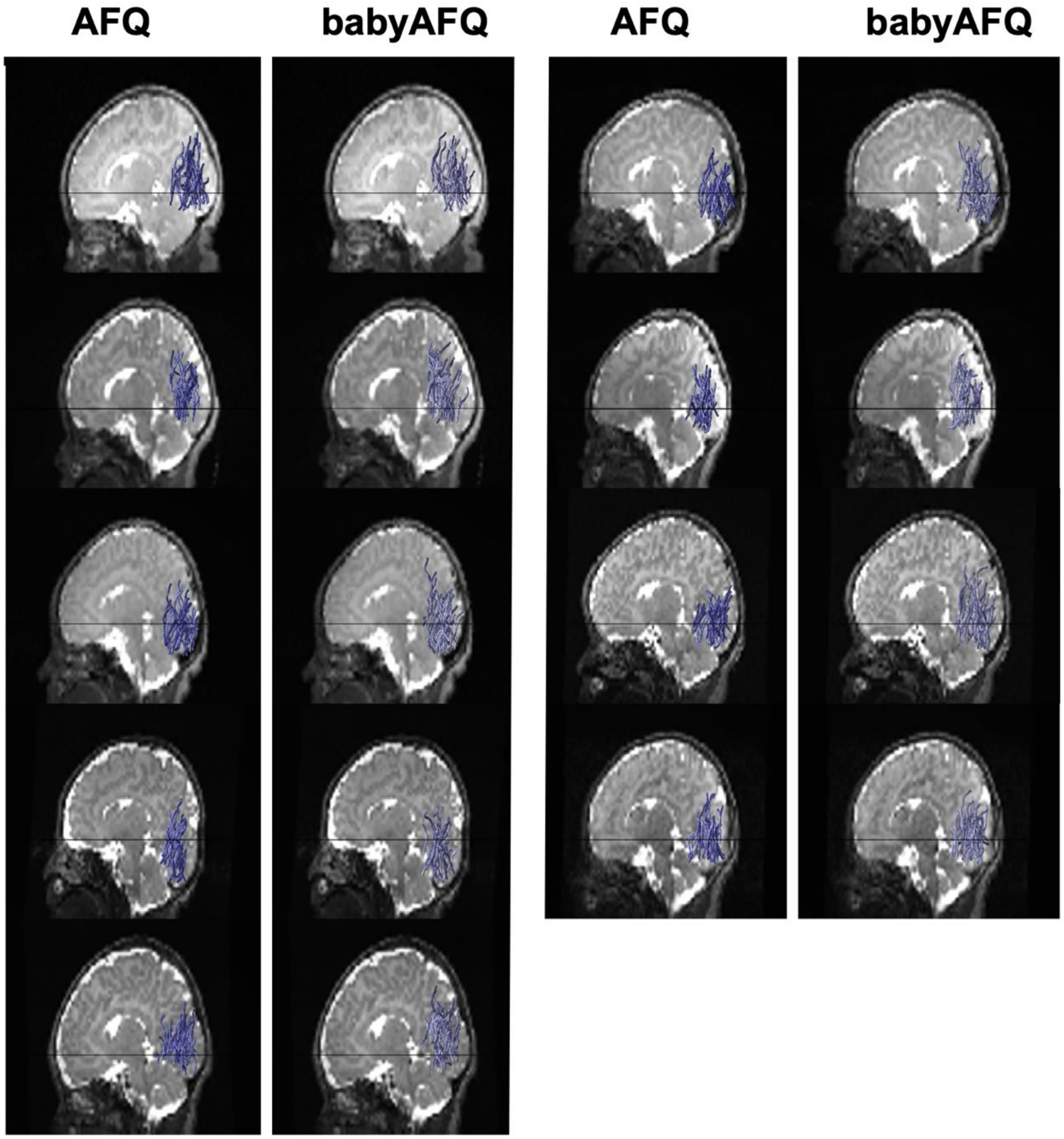

Right Vertical Occipital Fasciculus

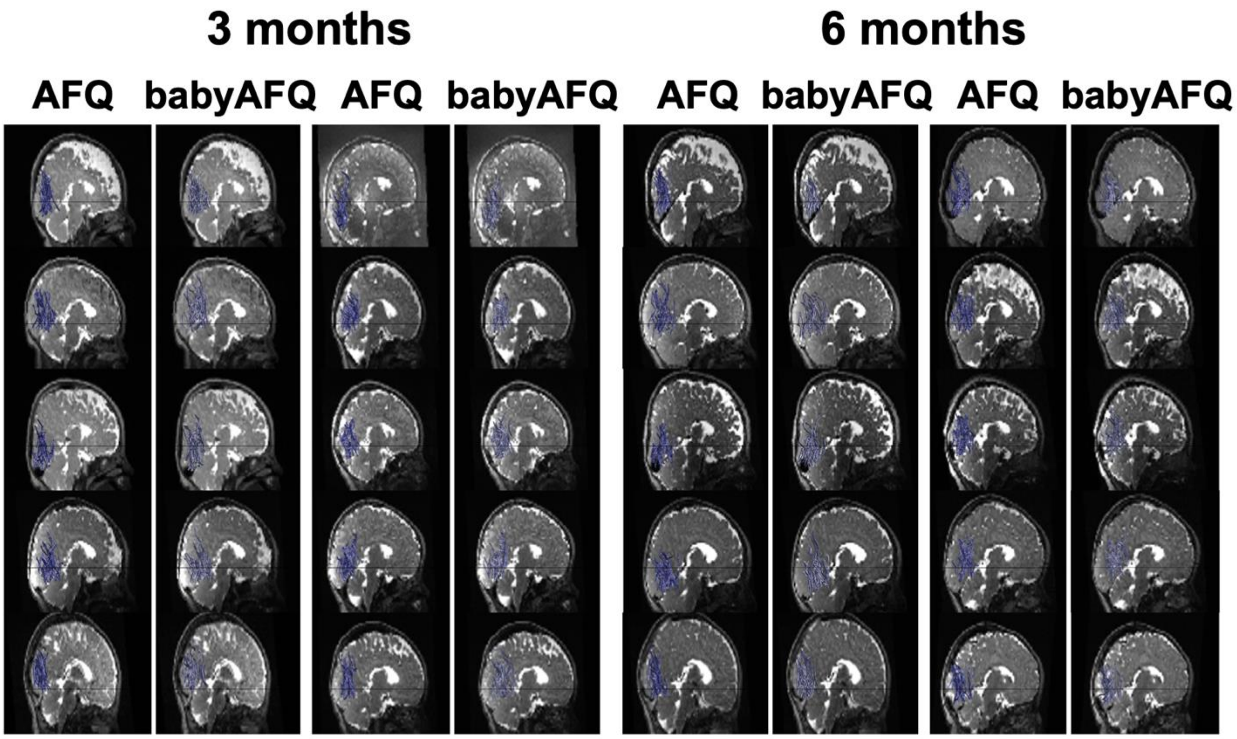

Supplement: Supplementary file 1 — Supplementary Information [file 41467_2022_28326_MOESM1_ESM.pdf]
