# Supplementary material for: White matter myelination during early infancy is linked to spatial gradients and myelin content at birth
Source: Nat Commun. 2022 Feb 22;13:997. doi: 10.1038/s41467-022-28326-4 (PMC8863985; doi:10.1038/s41467-022-28326-4)
Supplement: Supplementary file 3 — Reporting Summary [file 41467_2022_28326_MOESM3_ESM.pdf]

nature research

Corresponding author(s): Mareike Grotheer

Last updated by author(s): Dec 13, 2021

# Reporting Summary

Nature Research wishes to improve the reproducibility of the work that we publish. This form provides structure for consistency and transparency in reporting. For further information on Nature Research policies, see our Editorial Policies and the Editorial Policy Checklist.

## Statistics

For all statistical analyses, confirm that the following items are present in the figure legend, table legend, main text, or Methods section.

| n/a | Confirmed | |
|---|---|---|
| ☐ | ☒ | The exact sample size (*n*) for each experimental group/condition, given as a discrete number and unit of measurement |
| ☐ | ☒ | A statement on whether measurements were taken from distinct samples or whether the same sample was measured repeatedly |
| ☐ | ☒ | The statistical test(s) used AND whether they are one- or two-sided<br>*Only common tests should be described solely by name; describe more complex techniques in the Methods section.* |
| ☐ | ☒ | A description of all covariates tested |
| ☐ | ☒ | A description of any assumptions or corrections, such as tests of normality and adjustment for multiple comparisons |
| ☐ | ☒ | A full description of the statistical parameters including central tendency (e.g. means) or other basic estimates (e.g. regression coefficient) AND variation (e.g. standard deviation) or associated estimates of uncertainty (e.g. confidence intervals) |
| ☐ | ☒ | For null hypothesis testing, the test statistic (e.g. *F*, *t*, *r*) with confidence intervals, effect sizes, degrees of freedom and *P* value noted<br>*Give P values as exact values whenever suitable.* |
| ☒ | ☐ | For Bayesian analysis, information on the choice of priors and Markov chain Monte Carlo settings |
| ☒ | ☐ | For hierarchical and complex designs, identification of the appropriate level for tests and full reporting of outcomes |
| ☒ | ☐ | Estimates of effect sizes (e.g. Cohen's *d*, Pearson's *r*), indicating how they were calculated |

*Our web collection on statistics for biologists contains articles on many of the points above.*

## Software and code

Policy information about availability of computer code

| | |
|---|---|
| Data collection | No software was used for data collection, the infants were sleeping during the acquisition |
| Data analysis | The data were analyzed using open source software, specifically we used: mrDiffusion (v1.0, https://github.com/vistalab/vistasoft), AFQ (v1.2, https://github.com/yeatmanlab/AFQ), dsi-studio (downloaded in 2019, http://dsi-studio.labsolver.org/), MRtrix3 (RC3, https://www.mrtrix.org/), mrQ (v2.1, https://github.com/mezera/mrQ), infant FreeSurfer (downloaded in 2019, https://surfer.nmr.mgh.harvard.edu/fswiki/infantFS), Brain Extraction and Analysis Toolbox (iBEAT, v:2.0 cloud processing, https://ibeat.wildapricot.org/64–66), ITK-SNAP (downloaded in 2019, http://www.itksnap.org/), FSL (v6.0.2, https://fsl.fmrib.ox.ac.uk/), and ANTS (downloaded in 2019, http://stnava.github.io/ANTs/). Further, we developed a new toolbox for automatic fiber quantification in individual infants (babyAFQ) and made it openly available (https://github.com/yeatmanlab/AFQ/tree/babyAFQ/babyAFQ).<br>A demonstration of babyAFQ, including an example subject can be found here: https://figshare.com/s/456282406044bbb490ee.<br>Code to reproduce all figures is made available in GitHub (https://github.com/VPNL/babyWmDev) and on Zenodo (DOI: 10.5281/zenodo.5788646). |

For manuscripts utilizing custom algorithms or software that are central to the research but not yet described in published literature, software must be made available to editors and reviewers. We strongly encourage code deposition in a community repository (e.g. GitHub). See the Nature Research guidelines for submitting code & software for further information.



April 2020

## Data

Policy information about availability of data

All manuscripts must include a data availability statement. This statement should provide the following information, where applicable:

- Accession codes, unique identifiers, or web links for publicly available datasets
- A list of figures that have associated raw data
- A description of any restrictions on data availability

All data required to generate the main figures are provided as a Source Data file with this paper and is also made available in GitHub (https://github.com/VPNL/babyWmDev) and on Zenodo (DOI: 10.5281/zenodo.5788646).

# Field-specific reporting

Please select the one below that is the best fit for your research. If you are not sure, read the appropriate sections before making your selection.

☐ Life sciences ☒ Behavioural & social sciences ☐ Ecological, evolutionary & environmental sciences

For a reference copy of the document with all sections, see nature.com/documents/nr-reporting-summary-flat.pdf

# Behavioural & social sciences study design

All studies must disclose on these points even when the disclosure is negative.

| | |
|---|---|
| Study description | Here we investigated the myelination of white matter bundles during early infancy. To this end, we obtained longitudinal diffusion MRI and quantitative MRI measures of R1 in 0, 3 and 6 months-old human infants, and developed an automated method to identify white matter bundles and quantify their properties in each infant's brain. We find that R1 increases from newborns to 6-months-olds in all bundles. R1 development is nonuniform: there is faster development in white matter that is less mature in newborns, and along inferior-to-superior as well as anterior-to-posterior spatial gradients. |
| Research sample | 16 full-term and healthy infants (7 female) were recruited to participate in this study. Three infants provided no usable data because they could not stay asleep once the MRI sequences started and hence, we report data from 13 infants (6 female) across three timepoints: newborn (N=9; age: 8-37 days), 3 months (N=10; age: 79-106 days), and 6 months (N=10; age: 167-195 days). Two participants were re-invited to complete scans for their 6-months session that could not be completed during the first try. Both rescans were performed within 7 days and participants were still within age range for the 6-months timepoint.<br><br>The participant population was racially and ethnically diverse reflecting the population of the Bay Area, including two Hispanic, nine Caucasian, two Asian, and three multiracial participants. Six out of the 13 infants participated in MRI in all three timepoints (0, 3, 6 months). Due to the Covid-19 pandemic and restricted research guidelines, data acquisition was halted. Consequently, the remaining infants participated in either 1 or 2 sessions. |
| Sampling strategy | As in our previous pediatric samples (Golarai Nature Neuroscience 2007, Gomez Science 2017, Natu PNAS 2019) we planned to sample between 10 and 20 infants. This is a typical sample for our experiments as we perform analyses in individual infants' brain space. We sampled by convenience and recruited from the bay area in the vicinity of Stanford University.<br><br>Due to the COVID pandemic, and resulting restricted research guidelines, data collection was halted at the current sample of 16 participants. |
| Data collection | All included participants completed the multiple scanning protocols needed to obtain anatomical MRI, quantitative MRI (qMRI), and diffusion MRI (dMRI) data. Data were acquired at two identical 3T GE Discovery MR750 Scanners (GE Healthcare) and Nova 32-channel head coils (Nova Medical) located at Stanford University: (i) Center for Cognitive and Neurobiological Imaging (CNI) and (ii) Lucas Imaging Center. As infants have low weight, all imaging was done with first level SAR to ensure their safety. Study protocols for these scans were approved by the Stanford University Internal Review Board on Human Subjects Research.<br>Scanning sessions were scheduled in the evenings close in time to the infants' typical bedtime. Each session lasted between 2.5 – 5 hours including time to prepare the infant and waiting time for them to fall asleep. Upon arrival, caregivers provided written, informed consent for themselves and their infant to participate in the study. Before entering the MRI suite, both caregiver and infant were checked to ensure that they were metal-free and caregivers changed the infants into MR safe cotton onesies and footed pants provided by the researchers. The infant was swaddled with a blanket with their hands to their sides to avoid their hands creating a loop. During sessions involving newborn infants, an MR safe plastic immobilizer (MedVac, www.supertechx-ray.com) was used to stabilize the infant and their head position. Once the infant was ready for scanning, the caregiver and infant entered the MR suite. The caregiver was instructed to follow their child's typical sleep routine. As the infant was falling asleep, researchers inserted soft wax earplugs into the infant's ears. Once the infant was asleep, the caregiver was instructed to gently place the infant on a makeshift cradle on the scanner bed, created by weighted bags placed at the edges of the bed to prevent any side-to-side movement. Finally, to lower sound transmission, MRI compatible neonatal Noise Attenuators (https://newborncare.natus.com/products-services/newborn-care-products/nursery-essentials/minimuffs-neonatal-noise-attenuators) were placed on the infant's ears and additional pads were also placed around the infant's head to stabilize head motion.<br>An experimenter stayed inside the MR suite with the infant during the entire scan. For additional monitoring of the infant's safety and lack of motion, an infrared camera was affixed to the head coil and positioned for viewing the infant's face in the scanner. The researcher operating the scanner monitored the infant via the camera feed, which allowed for the scan to be stopped immediately if the infant showed signs of waking or distress. This setup also allowed tracking the infant's motion; scans were stopped and repeated if there was excessive head motion. To ensure scan data quality, in addition to real-time monitoring of the infant's motion via an |

| | |
|---|---|
| | infrared camera, MR brain image quality was also assessed immediately after acquisition of each sequence and repeated if necessary. The experimenters were not blinded to the study design and hypotheses. |
| Timing | Data collection began in spring 2019 and was halted in March 2020 due to the Covid-19 pandemic and resulting restricted research guidelines |
| Data exclusions | Three infants provided no usable data because they could not stay asleep once the MRI sequences started. No data was excluded from infants that completed all required scans. |
| Non-participation | No participants dropped out or declined participation |
| Randomization | Randomization was not applicable, as we had only one participant group and used a longitudinal design |

# Reporting for specific materials, systems and methods

We require information from authors about some types of materials, experimental systems and methods used in many studies. Here, indicate whether each material, system or method listed is relevant to your study. If you are not sure if a list item applies to your research, read the appropriate section before selecting a response.

## Materials & experimental systems

| n/a | Involved in the study | |
|---|---|---|
| ☒ | ☐ | Antibodies |
| ☒ | ☐ | Eukaryotic cell lines |
| ☒ | ☐ | Palaeontology and archaeology |
| ☒ | ☐ | Animals and other organisms |
| ☐ | ☒ | Human research participants |
| ☒ | ☐ | Clinical data |
| ☒ | ☐ | Dual use research of concern |

## Methods

| n/a | Involved in the study | |
|---|---|---|
| ☒ | ☐ | ChIP-seq |
| ☒ | ☐ | Flow cytometry |
| ☐ | ☒ | MRI-based neuroimaging |

## Human research participants

Policy information about studies involving human research participants

| | |
|---|---|
| Population characteristics | See above |
| Recruitment | Expectant mothers and their infants were recruited from the San Francisco Bay Area using social media platforms. We performed a two-step screening process for expectant mothers. First, mothers were screened over the phone for eligibility based on exclusionary criteria designed to recruit a sample of typically developing infants and second, eligible expectant mothers were screened once again after giving birth. Exclusionary criteria for expectant mothers were as follows: recreational drug use during pregnancy, significant alcohol use during pregnancy (more than 3 instances of alcohol consumption per trimester; more than 1 drink per occasion), lifetime diagnosis of autism spectrum disorder or a disorder involving psychosis or mania, taking prescription medications for any of these disorders during pregnancy, insufficient English ability to understand study instructions, and learning differences that would preclude participation in the study. Exclusionary criteria for infants were: preterm birth (<37 gestational weeks), low birthweight (<5 lbs 8 oz), small height (<18 inches), any congenital, genetic, and neurological disorders, visual problems, complications during birth that involved the infant (e.g., NICU stay), history of head trauma, and contraindications for MRI (e.g., metal implants).<br><br>The resulting participant population was racially and ethnically diverse reflecting the population of the Bay Area, including two Hispanic, nine Caucasian, two Asian, and three multiracial participants. We are not aware of any self-selection biases or other biases in our sample. |
| Ethics oversight | Study protocols were approved by the Stanford University Internal Review Board on Human Subjects Research. Upon arrival, caregivers provided written, informed consent for themselves and their infant to participate in the study. |

Note that full information on the approval of the study protocol must also be provided in the manuscript.

## Magnetic resonance imaging

### Experimental design

| | |
|---|---|
| Design type | anatomical data only, data was aquired during natural sleep |
| Design specifications | n/a |
| Behavioral performance measures | baby stays asleep and is still |

## Acquisition

Imaging type(s): diffusion, structural and quantitative MRI

Field strength: 3T

Sequence & imaging parameters: Anatomical MRI: T2-weighted images were acquired and used for tissue segmentations. T2-weighed image acquisition parameters: TE=124 ms; TR = 3650ms; echo train length = 120; voxel size = 0.8mm3; FOV=20.5cm; Scan time: 4 min and 5 sec.
Quantitative MRI: An inversion-recovery EPI (IR-EPI) sequence was used to estimate relaxation time (R1) at each voxel. Spoiled-gradient echo images (SPGRs) were used together with the EPI sequence to generate whole-brain synthetic T1-weighted images. We acquired 4 SPGRs whole brain images with different flip angles: α = 4, 10, 15, 20 degrees; TE=3ms; TR =14ms; voxel size=1mm3; number of slices=120; FOV=22.4cm; Scan time: 4 times ~5 minutes. We also acquired multiple inversion times (TI) in the IR-EPI using a slice-shuffling technique: 20 TIs with the first TI=50ms and TI interval=150ms as well as a second IR-EPI with reverse phase encoding direction. Other acquisition parameters were: voxel size=2mm3; number of slices=60; FOV=20cm; in-plane/through-plane acceleration=1/3; Scan time=two times 1:45 min.
Diffusion MRI: We obtained dMRI data with the following parameters: multi-shell, #diffusion directions/b-value = 9/0, 30/700, 64/2000; TE = 75.7 ms; TR=2800ms; voxel size = 2mm3; number of slices=60; FOV=20cm; in-plane/through-plane acceleration = 1/3; Scan time: 5:08 min. We also acquired a short dMRI scan with reverse phase encoding direction and only 6 b=0 images (scan time 0:20 min).

Area of acquisition: Whole Brain

Diffusion MRI: ☒ Used ☐ Not used

Parameters: We obtained dMRI data with the following parameters: multi-shell, #diffusion directions/b-value = 9/0, 30/700, 64/2000; TE = 75.7 ms; TR=2800ms; voxel size = 2mm3; number of slices=60; FOV=20cm; in-plane/through-plane acceleration = 1/3; Scan time: 5:08 min. We also acquired a short dMRI scan with reverse phase encoding direction and only 6 b=0 images (scan time 0:20 min).

## Preprocessing

Preprocessing software: dMRI preprocessing was performed in accordance with recent work from the developing human connectome project, using a combination of tools from MRtrix3 (github.com/MRtrix3/mrtrix3) and mrDiffusion (http://github.com/vistalab/vistasoft). We (i) denoised the data using a principal component analysis, (ii) used FSL's top-up tool (https://fsl.fmrib.ox.ac.uk/) and one image collected in the opposite phase-encoding direction to correct for susceptibility-induced distortions, (iii) used FSL's eddy to perform eddy current and motion correction, whereby motion correction included outlier slice detection and replacement and (iv) performed bias correction using ANTs. The preprocessed dMRI images were registered to the whole-brain T2-weighted anatomy using whole-brain rigid-body registration and alignment quality was checked for all images. dMRI quality assurance was also performed. Across all acquisitions, less than 5% ± 0.72% of dMRI images were identified as outliers by FSL's eddy tool.

Normalization: No normalization; data were analyzed in each individual's native brain space at each time point

Normalization template: Data were not normalized

Noise and artifact removal: We (i) denoised the data using a principal component analysis, (ii) used FSL's top-up tool (https://fsl.fmrib.ox.ac.uk/) and one image collected in the opposite phase-encoding direction to correct for susceptibility-induced distortions, (iii) used FSL's eddy to perform eddy current and motion correction, whereby motion correction included outlier slice detection and replacement and (iv) performed bias correction using ANTs.

Volume censoring: n/a

## Statistical modeling & inference

Model type and settings: We modeled R1 and MD development using mixed linear models. First, we modeled mean R1/MD development within each white matter bundle using linear mixed models (LMMs) with age as predictor and a random intercept (estimated R1/MD at birth) for each individual. We used model comparison (likelihood ratio tests) to determine that LMMs allowing different slopes for each individual do not better explain the data compared to LMMs using a single slope across individuals. We then determined the rate of R1/MD development across the length of each bundle by fitting LMMs that relate R1/MD to age at each node (one LMM per bundle; random intercepts for each individual as above). Finally, we evaluated the relationship between the slope of R1/MD development and the measured R1/MD in newborns as well as the spatial location in the brain at nonoverlapping positions (every 10th node) along all bundles (LMM relating R1/MD slope to measured R1/MD in newborns, as well as, the nodes |x|,y,z coordinates, random intercepts for each bundle).

Effect(s) tested: Effect of using babyAFQ rather than AFQ (repeated measures Anova)
Effect of age on R1/MD across and along bundles (LMMs see above)
Relationship between R1 /MD at birth and R1/MD development rate
Relationship between spatial location in the brain and R1/MD development rate

Specify type of analysis: ☐ Whole brain ☒ ROI-based ☐ Both

| | |
|---|---|
| Anatomical location(s) | 26 white matter bundles of the brain were automatically identified using newly developed babyAFQ |
| Statistic type for inference (See Eklund et al. 2016) | n/a |
| Correction | n/a |

## Models & analysis

| n/a | Involved in the study |
|---|---|
| ☒ | ☐ Functional and/or effective connectivity |
| ☒ | ☐ Graph analysis |
| ☒ | ☐ Multivariate modeling or predictive analysis |
